# Homotrimer cavin1 interacts with caveolin1 to facilitate tumor growth and activate microglia through extracellular vesicles in glioma

**DOI:** 10.7150/thno.45688

**Published:** 2020-05-17

**Authors:** Lin Wang, Chao Yang, Qixue Wang, Qi Liu, Yunfei Wang, Junhu Zhou, Yansheng Li, Yanli Tan, Chunsheng Kang

**Affiliations:** 1 Department of Neurosurgery, Tianjin Medical University General Hospital, Tianjin 300052, China;; 2 Tianjin Neurological Institute, Key Laboratory of Post-neurotrauma Neuro-repair and Regeneration in Central Nervous System, Ministry of Education and Tianjin City, Tianjin 300052, China;; 3 State Key Laboratory of Medicinal Chemical Biology, Key Laboratory of Functional Polymer Materials of Ministry of Education, College of Chemistry, Nankai University, Tianjin 300071, China;; 4 Department of Pathology, Affiliated Hospital of Hebei University, Baoding 071000, China;; 5 Department of Pathology, Hebei University Medical College, Baoding 071000, China.

**Keywords:** Cavin1-Caveolin1 interaction, extracellular vesicles, glioma, microglia

## Abstract

**Background**: Intercellular communication via extracellular vesicles (EVs) plays a critical role in glioma progression. However, little is known about the precise mechanism regulating EV secretion and function. Our previous study revealed that Cavin1 was positively correlated with malignancy grades of glioma patients, and that overexpressing Cavin1 in glioma cells enhanced the malignancy of nearby glioma cells via EVs.

**Methods**: The current study used bioinformatics to design a variant Cavin1 (vCavin1) incapable of interacting with Caveolin1, and compared the effects of overexpressing Cavin1 and vCavin1 in glioma cells on EV production and function.

**Results**: Remarkably, our results indicated that Cavin1 expression enhanced the secretion, uptake, and homing ability of glioma-derived EVs. EVs expressing Cavin1 promoted glioma growth *in vitro* and *in vivo*. In addition, Cavin1 expressing murine glioma cells recruited and activated microglia via EVs. However, vCavin1 neither was loaded onto EVs nor altered EV secretion and function.

**Conclusion**: Our findings suggested that Cavin1-Caveolin1 interaction played a significant role in regulating production and function of glioma-EVs, and may act as a promising therapeutic target in gliomas that express high levels of Cavin1.

## Introduction

Communication between glioma cells, as well as between glioma cells and various surrounding cells in the microenvironment enables glioma progression and resistance to therapy. The communication patterns are multiple, including direct interactions (e.g., gap junctions and connecting nanotubes), extracellular vesicles (EVs), soluble factors such as cytokines and chemokines, and mechanotransduction 1. Mediation of cell communication by EVs within the tumor microenvironment is rapidly emerging as a hotspot which is attracting much attention. Small EVs of 40~150 nm in diameter originate from the endosomal system via invagination of the plasma membrane and early endosomes, which then mature into multivesicular bodies (MVBs) 2, 3. Glioma-derived EVs containing proteins, lipids, RNA and DNA are exchanged between tumor cells or delivered to normal cells, such as microglia, endothelial cells and astrocytes, thereby enabling the transfer of malignant phenotypes within the microenvironment and promoting glioma cell viability and proliferation, angiogenesis, and chemo-resistance 4-10.

Cavin1, also known as Polymerase I and transcript release factor (PTRF), is a caveolar-associated protein. Together with caveolins, it plays an essential role in the formation and function of caveolae 11, 12. Caveolae are flask-shaped plasma membrane invaginations of 50-100 nm diameter that play a crucial role in various cellular processes, such as signal transduction, membrane trafficking and endocytosis 13-15. As one form of endocytosis, caveolae bud off from the plasma membrane, transition to Early Endosome Antigen 1 (EEA1)-positive early endosomes or recycle back to the plasma membrane 16. Abnormal caveolae are related to a series of malignant diseases. Caveolar components Cavin1, Caveolin1, and CD36 have all been reported as prognostic biomarkers in patients with solid tumors 17-22. In particular, dysregulated Cavin1 has been identified as being associated with prostate cancer, Ewing's sarcoma and lung cancer 19, 23, 24. A previous study of ours revealed that Cavin1 was involved in EV mediated communication among glioma cells 18. Cavin1 expression in glioma cells increased the malignancy of nearby cells via EVs *in vitro* and *in vivo*, and Cavin1 was positively correlated with tumor grades and poor prognoses in glioma patients 18. However, the effects exerted by Cavin1-overexpression on glioma EVs as well as molecular mechanisms underlying such effects remain unclear.

Caveolins are the main scaffolding proteins of caveolae. Caveolin1 and Caveolin3 are required for the formation of caveolae in non-muscle and muscle tissues, respectively 25, 26. Caveolin2, the expression distribution of which was very similar to that of Caveolin1, is reportedly dispensable for caveolae formation *in vivo* 27. FRET experiments detected close proximity between Cavin1 and Caveolin1 at the cell surface 28. It is generally reported that Cavin1 recruitment to caveolae and binding to Caveolin1 are critical for caveolar genesis and function. However, whether there is direct interaction between Cavin1 and Caveolin1 remains controversial, and the role interaction between Cavin1 and Caveolin1 plays in glioma biology has not been clarified.

Here, we report on an essential role played by Cavin1-Caveolin1 binding in regulating glioma EV secretion and function. The variant Cavin1 (vCavin1) was constructed by fusing a short peptide “TAT” rich in positively charged amino acids (GYGRKKRRQRRRG) to the N-terminus of Cavin1 to hinder its interaction with Caveolin1. Molecular dynamics and protein docking simulation showed that the combination of vCavin1 and Caveolin1 was unstable, which was confirmed via immunoprecipitation assays and fluorescence confocal analyses. Overexpressed Cavin1 in glioma cells could be loaded into EVs, whereas overexpressed vCavin1 was not detected in EVs, which indicated the importance of the Caveolin1-association for Cavin1 recruitment into EVs. Compared with the vCavin1, Cavin1 in glioma cells significantly increased EV secretion, uptake and homing ability. In addition, glioma EVs expressing Cavin1 enhanced proliferation of nearby glioma cells and exerted recruiting and activating effects on microglia.

## Materials and Methods

### Structure prediction

The amino acid sequence of Cavin1 (NP_036364.2) was obtained from the National Center for Biotechnology Information (NCBI), and the sequence of vCavin1 was obtained by modifying the Cavin1 sequence. ProtParam (http://web.expasy.org/protparam/) and ProtScale tools (http://web.expasy.org/protscale/) were used to predict physicochemical and hydrophobic properties of Cavin1. Three-dimensional (3D) structural models of Cavin1 and vCavin1 were generated using the I-TASSER online prediction server (https://zhanglab.ccmb.med.umich.edu/I-TASSER/). Briefly, I-TASSER automatically identified templates in the Protein Data Bank database through LOMETS and automatically generated templates with high similarity to the target protein sequence in a low-to-high order. Following sequence alignment, homology modeling was performed and the structure was optimized using Gromacs4.6 kinetic software. Physicochemical parameters of the model were evaluated by Ramachandran and ERRAT plots.

### Molecular dynamics simulation

Molecular dynamics simulation of Caveolin1-Cavin1 membrane protein was performed using Gromacs 4.6.3 software. GROMOS 53A6 force field was used for protein and the SPC model was used for water molecules. The system was optimized by the steepest descent method and the conjugate gradient method to achieve the best state. In order to adapt the system to the simulated environment, it was subjected to constant temperature (NVT) and constant pressure (NPT) ensemble balance. During the balancing process, temperature coupling was achieved using the V-rescale method (thermal coupling time constant: 0.1 ps). Next, the Caveolin1-Cavin1 membrane protein molecular system was simulated for 10 ns using the leapfrog algorithm. The integration step was set to 2 fs, the long-range electrostatic interaction was processed by PME algorithm and the short-range Coulomb truncation radius, and the truncation radius for calculating van der Waals was both set at 1.2 nm. In the system, the periodic boundary condition was used in all directions, and the LINCS algorithm was used to constrain the bond length of protein and lipid molecules. Simulation results were analyzed via Gromacs 4.6 and visualized by VMD.

### Docking simulations

Docking simulations between Caveolin1 and Cavin1/vCavin1 were performed using ZDock to predict all possible interaction patterns. The 82~101 residue of the receptor protein Caveolin1 is a structural region for scaffold proteins, and the 102~134 residue is a region for transmembrane structures. Other proteins are not bound to the scaffold structure region and the transmembrane region. Therefore, the 82-134 residue of Caveolin1 was prevented from interacting with the ligand, and all possible spatial conformations and interaction patterns were fully searched. The conformation with the lowest energy was selected for visualization analysis using PyMol v1.60.

### Cell culture

Human glioblastoma cell lines U87MG, LN229, murine glioma cell line GL261 and murine microglia BV2 were purchased from the American Type Culture Collection (ATCC, Manassas; Virginia, US). The primary cell line, TBD0220, was derived from a GBM patient who underwent a surgery at Hebei University Affiliated Hospital. U87, LN229 and GL261 were maintained in Dulbecco's modified Eagle's medium (DMEM, Gibco) supplemented with 10% EV-depleted fetal bovine serum (FBS). U87 and GL261 cells were transduced with lentiviruses encoding the eGFP-Cavin1 or eGFP-vCavin1 fusion protein (Genechem Co.LTD.; Shanghai, China) followed by puromycin treatment for 1 week.

### Detection of contamination

The cells were tested for mycoplasma contamination using a MycoAlert Mycoplasma Detection Kit (LT07-218; Lonza; USA) and for endotoxin contamination using a Kinetic-QCL™ Kinetic Chromogenic LAL Assay (50-650NV; Lonza; USA) according to the manufacturers' instructions.

### Scanning electron microscopy (SEM) and Transmission electron microscopy (TEM)

An equal number of U87-eGFP, U87-C, and U87-vC cells (1×10^6^/dish) were seeded on culture dishes and cultured for 24 h (to avoid the effects of different cell proliferation rates after 24 h on EVs production). For SEM, the cells were fixed overnight with 2.5% glutaraldehyde at 4 °C and rinsed with 0.1 M phosphate buffered saline thrice. After being post-fixed in 1% osmium tetroxide for 60 min, the samples were dehydrated via an ascending ethanol gradient and dried with hexamethyldisilazane. The samples were then sputtered with gold-palladium and examined under a scanning electron microscope (SEM, JSM-7900F; JEOL; Japan) operating at 3 kV. For TEM, cells were fixed with 2.5% glutaraldehyde, post-fixed with 1% osmium tetroxide, dehydrated in graded ethanol, and embedded in epoxy resin. Ultra-thin slices were sectioned using an ultramicrotome, stained with 2% uranyl acetate and lead citrate and imaged under a JEOL JEM-1400 TEM at an operating voltage of 80 kV.

### Immunoprecipitation

Whole-cell lysates of U87, U87-C, U87-vC, GL261, GL261-C, and GL261-vC cells were prepared using lysis buffer and centrifuged, following which the supernatants were mixed with 1.0 µg of rabbit IgG, together with 20 µL of Protein A/G PLUS-Agarose (sc-2003; Santa Cruz Biotechnology; CA, USA) and incubated at 4 °C for 30 min to remove nonspecific proteins. After centrifugation, protein concentration of the supernatants was determined via a BCA assay and adjusted to 1 mg/mL. The lysates was incubated with 5 μL of anti-GFP antibodies (ab290; Abcam; UK) or control IgG overnight at 4 °C with rotation and then 20 µL of Protein A/G Agarose at 4 ℃ for 6 h. Immunoprecipitates were then collected via centrifugation and washed with RIPA buffer 4 times.

### Western blot

Cells or EVs were lysed using RIPA buffer supplemented with protease and phosphatase inhibitors. Lysates were collected and centrifuged at 14,000 g for 15 min at 4 °C. The protein content of the supernatants was measured via a BCA assay. Each sample (containing 40 μg protein) was separated on 10% or 15% SDS-acrylamide gels by electrophoresis, and transferred onto PVDF membranes. The membranes were blocked with 5% bovine serum albumin (BSA) in PBST for 1 h, incubated with primary antibodies against Cavin1 (18892-1-AP; Proteintech; Wuhan, China), Caveolin1 (MAB5736; R&D; USA), Caveolin2 (410700; Life Technologies; USA), GAPDH (ab8245; Abcam; UK), CD63 (25682-1-AP; Proteintech), Alix (#92880; Cell Signaling Technology; USA) and CD81 (66866-1-Ig; Proteintech) for 12 h at 4 °C, followed by incubation with HRP conjugated secondary antibodies for 1 h at room temperature. Protein bands were visualized using enhanced chemiluminescence (ECL) substrate and band intensities were quantified with Image J.

### EV isolation and characterization

In view of increased proliferation of U87-C compared with that of U87-eGFP and U87-vC starting from 48 h post-seeding (Supplementary Fig. 5E), we collected supernatant at 24 h post-seeding to ensure that EVs of different groups were derived from an equal number of cells. U87-eGFP, U87-C, and U87-vC were respectively seeded in 100 mm culture dishes at 1×10^6^ cells per dish and cultured for 24 h in media supplemented with 10% EV-depleted FBS. Next, the conditioned media were collected and sequential centrifugations were performed to isolate EVs. In brief, differential centrifugations at 800 g (10 min, 4 ℃) and 2,000 g (20 min, 4 ℃) to discard dead cells and cellular debris, at 10,000 g (30 min, 4 ℃) to eliminate microvesicles, and at 100,000 g (70 min, 4 ℃) to pellet EVs. EV pellets were then washed in PBS, centrifuged at 100,000 g for 70 min and resuspended in 200 µL PBS.

Size distribution and quantity of particles of isolated EVs was measured using the NanoSight system (NS300; Malvern instruments; UK). EV samples were diluted in PBS at an optimal concentration and each sample was captured for 50 s (number of captures: 3) with the detection threshold set at 5. Recorded videos were then analyzed using NTA software 3.2.

The amount of protein in EV samples was measured using a BCA Protein Assay Kit (Solarbio) according to the manufacturer's protocols.

The morphology of EV samples was visualized using transmission electron microscopy. EV samples were pipetted onto carbon-coated EM grids, stained with 1% uranyl acetate, air-dried at 26 ℃ and viewed under a transmission electron microscope (JEM-1400; JEOL) operating at 100 kV.

### Flow cytometric analysis of the cellular uptake of EVs

LN229 cells were incubated at 37 °C with 0.5 mg/mL of Cy5-labeled U87-EVs, U87-C-EVs, and U87-vC-EVs, respectively, for 15 min and 1 h. Cells were harvested, washed twice with PBS, and resuspended in PBS. Cy5 fluorescence intensity within the cells was analyzed using flow cytometry (Guava easyCyte 8HT; Millipore; USA). GeoMean fluorescence intensities (MFI) of Cy5 were quantified using FlowJo V10 software.

### Cell proliferation assay

Glioma cell proliferation was measured using a Cell Counting Kit-8 assay (CCK-8, Dojin; Japan) according to the manufacturer's instruction. Briefly, 1 × 10^3^ cells were seeded in 96-well plates. At 24 h, 48 h, 72 h, 96 h, 120 h, and 144 h post seeding, cells were incubated with CCK-8 solution (10 μL/well) for 2 h at 37 °C. Optical density (OD) values were measured at 450 nm with a microtiter plate reader (BioTek Synergy^tm^ 2; Vermont, USA). Relative cell growth was expressed as a ratio of the OD value at each indicated time point to the OD value at 0 h. The assay was repeated thrice.

### Orthotopic glioma mouse model

All animal experiments were performed in accordance with protocols approved by the Animal Ethical and Welfare Committee (AEWC) of Tianjin Medical University. Five-week-old female nude mice or C57/BL6 mice were used to generate intracranial orthotopic LN229/U87 or GL261 gliomas, respectively. LN229 were transduced with lentivirus stably expressing firefly luciferase and red fluorescence protein (RFP). U87 human glioma cells expressing eGFP, eGFP-Cavin1, or eGFP-vCavin1 (2.5×10^5^) were respectively mixed with an equal number of LN229-RFP-luc (2.5×10^5^) in PBS and injected 3 mm deep into the right hemisphere of the brain in nude mice. Nude mice were randomly separated into 3 groups: (i) LN229-RFP-luc+U87-eGFP, (ii) LN229-RFP-luc+U87-C, (iii) LN229-RFP-luc+U87-vC. Body weight was measured every other day and tumor growth was monitored at 7, 14, 21, and 28 d using a bioluminescence IVIS® imaging system (Xenogen, Almeda; CA, USA) (n = 8). GL261 murine glioma cells expressing eGFP, eGFP-Cavin1 or eGFP-vCavin1 (6×10^5^) were implanted orthotopically in C57/BL6 mice and tumors were allowed to grow for 35 d before the euthanization of the mice.

### Biodistribution of EVs in mouse brain

Nude mice bearing orthotopic glioma xenografts (TBD0220) were used to evaluate EV distribution in the brain. U87, U87-C, and U87-vC-derived EVs were labeled with Cy5.5 NHS ester (A8103; ApexBio Technology; USA) and injected into the tail vein of mice at a concentration of 1 mg/mL (200 μL per mouse). Cy5.5 fluorescence in mice brains was monitored at 2, 6, and 24 h post injection using IVIS Spectrum *in vivo* imaging system (Xenogen) (n = 9). Subsequently, the mice were sacrificed 24 h post injection, perfused and the brains were immediately isolated and analyzed by the IVIS system to evaluate Cy5.5 fluorescence and tumor bioluminescence (n = 4). Finally, the brains were fixed in 4% paraformaldehyde for confocal microscopy (n = 5).

### Confocal and two-photon microscopy

On day 21 after implantation of LN229-RFP-luc/U87-eGFP, LN229-RFP-luc/U87-C and LN229-RFP-luc/U87-vC, mice (n = 4) were sacrificed and perfused transcardially with cold PBS and 4% PFA in PBS. The brains were then post-fixed in 4% PFA, dehydrated successively in 20% and 30% sucrose, embedded in optimal cutting temperature (OCT) compound (Sakura; Tokyo, Japan), frozen at -80 ℃ and sliced into coronal sections (8 μm and 80 μm). For 3D Z-stack imaging, 80 μm-thick sections were viewed using an Olympus FV-1000MPE two-photon microscope (Olympus; Japan) equipped with a water-immersion objective (XLPlan N 25×/0.05 W MP). The 80 μm-deep stacks were acquired with a 1.6 μm step depth and analyzed using Olympus FV31S-SW.

Next, 8 μm frozen sections or cells grown on glass coverslips were fixed with 4% PFA for 30 min, permeabilized with 0.2% Triton X-100 in PBS, blocked with 5% BSA for 1 h and incubated with primary antibodies against Caveolin1 (MAB5736; R&D), Caveolin2 (410700; Life technologies), EEA1 (66218-1-Ig; Proteintech), and Cavin1 (18892-1-AP; Proteintech) for 12 h at 4 ℃. Sections were then incubated with Alexa Fluor 488-, or 594-conjugated secondary antibodies (Life Technologies) for 1 h, followed by counterstaining with DAPI (C0060; Solarbio; Beijing, China). Images were captured using a confocal fluorescence microscope (Olympus, FluoView 1200; Tokyo, Japan).

### Migration assay

A BV2 migration assay was performed using transwell chambers (8 μm pore size, PI8P01250, Millipore) on 24-well plates. BV2 (2×10^4^ cells/well) were suspended in low-serum (2% FBS) medium and seeded in the upper chamber. EV-depleted medium (10% FBS) supplemented with GL261-EVs, GL261-C-EVs, and GL261-vC-EVs (0.5 mg/mL), respectively, was placed on the lower chamber. Medium containing no EVs was used as the control. Following 48 h incubation in 37 ℃, the migrated cells on the lower surface were fixed with methanol and stained with crystal violet. Migration ability was expressed as the mean number of migrated cells per 1×10^4^ μm^2^.

### Immunohistochemical analysis

Nude mice at 21 d post injection with LN229-RFP-luc/U87-C (n = 5) and C57BL/6 mice at 35 d post injection with GL261-C (n = 6) were sacrificed and transcardially perfused with PBS and 4% PFA. The brains were post-fixed in 4% PFA, embedded in paraffin, and sliced into 5 µm-thick coronal sections. Next, the sections were deparaffinized with xylene, and rehydrated with a descending ethanol gradient, followed by antigen retrieval. The sections were then treated with 0.3% H_2_O_2_ for 20 min to inhibit endogenous peroxidase, and incubated with 5% goat serum for 30 min. Next, sections were incubated with indicated primary antibodies against Ki67 (MA5-15525; Invitrogen), CD68 (ab125212; Abcam), CD86 (14-0862-82; eBioscience; CA, USA), MHC Ⅱ (Ab180779; Abcam), CD206 (PA5-46994; Invitrogen) and CD163 (PA5-78961; Invitrogen) for 12 h at 4 ℃, washed with PBS and incubated with biotinylated secondary antibodies at 37 ℃ for 60 min. After washing with PBS, the sections were stained with diaminobenzidine (DAB) and counterstained with haematoxylin.

### Statistical analysis

GraphPad Prism 7 was used for statistical analysis and graphing. Unpaired Student's t-test was used for comparison between two groups, and one-way ANOVA followed by LSD test was applied for multi-group (> 2 groups) comparisons. Data were expressed as the mean ± SEM (ns represents p > 0.05; * = p < 0.05, ** = p < 0.01, *** = p < 0.001 and **** = p < 0.0001).

## Results

### The N-terminus of vCavin1 differs in structure and electrostatic surface properties from Cavin1's N-terminus

**Figure 1:**
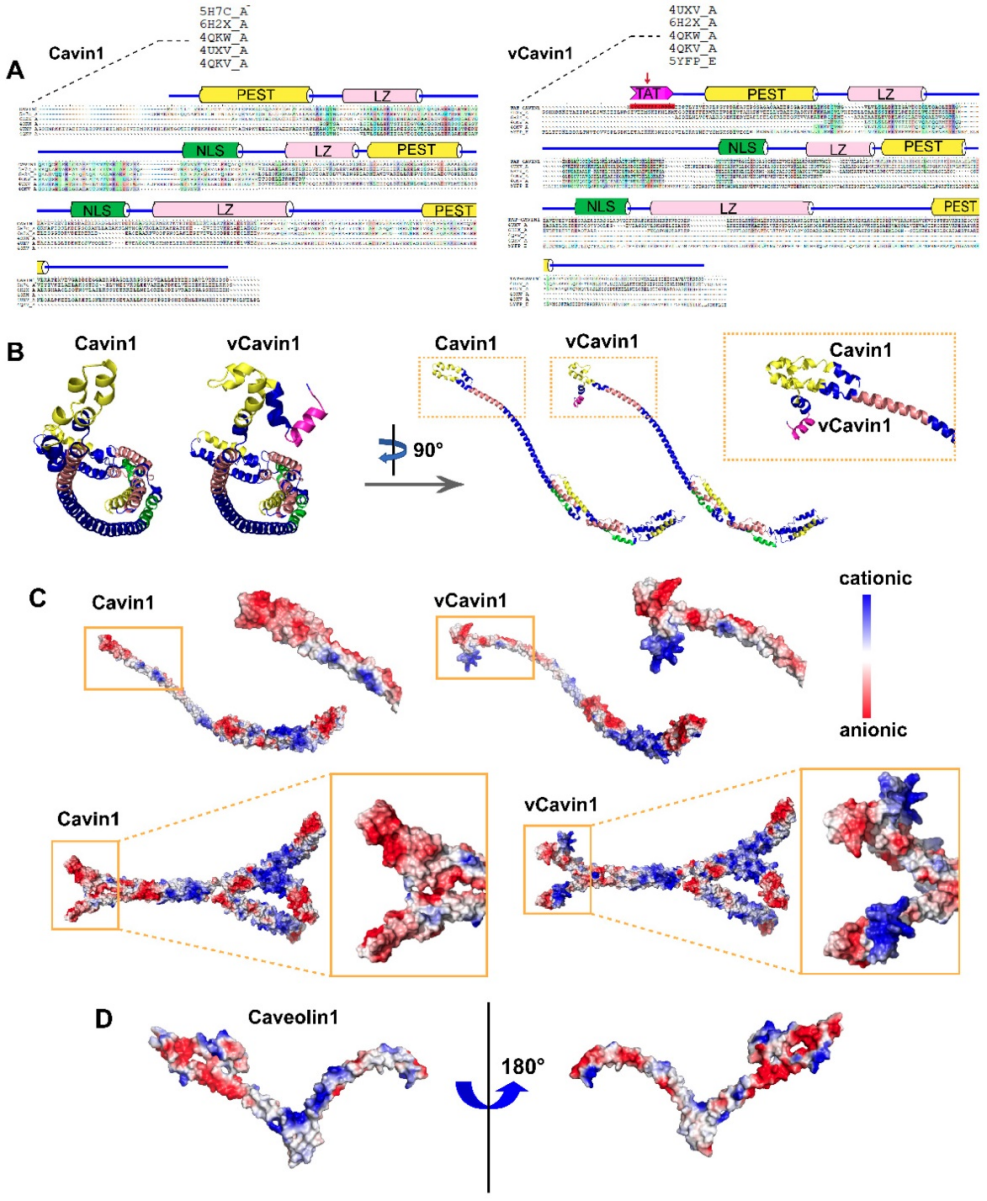
** Construction of 3D structures of Cavin1, vCavin1 and Caveolin1 protein.** (A) Sequence alignment of human Cavin1 and vCavin1 with high homology templates, respectively. Corresponding domains are shown at the top. The fused TAT short peptide is highlighted by a red arrow. PEST: Pro-Glu-Ser-Thr sequence, LZ: leucine-zipper domain, NLS: nuclear localization sequence. (B) Front and side views of 3D structures of Cavin1 and vCavin1 monomer. The N-terminuses of Cavin1 and vCavin1 were different in structure and orientation. (C) Electrostatic surface view of the monomer and trimer of Cavin1 and vCavin1. Different electrostatic potential in the N-terminus of Cavin1 and vCavin1 monomer or inside the telechelic cavity of Cavin1 and vCavin1 trimer. Blue: cationic; white: electroneutral; red: anionic. (D) Electrostatic surface view of Caveolin1 monomer. The N-terminus of Caveolin1 shows an electrostatic potential matched to the inner surface of the telechelic cavity of Cavin1.

Number and proportion of amino acids and physicochemical properties of Cavin1 were summarized in Table S1 and Table S2, respectively. I-TASSER was used for homology modeling to obtain accurate three-dimensional structural models of Cavin1 and vCavin1. I-TASSER automatically identified templates in the Protein Data Bank database through LOMETS and automatically generated templates with a high similarity to the target protein sequence in a low-to-high order. The templates with higher sequence identities and longer aligned lengths were 5H7C, 1VW1, 4QKW, 4UXV and 4QKV for Cavin1 (Table 1), and 4UXVA, 6H2XA, 4QKWA, 4QKVA, and 5YFPE for vCavin1 (Table 2). Among these, 5H7C, 1VW1 and 4UXV showed poor homology but high coverage, while 4QKW and 4QKV showed lower coverage but higher homology. Therefore, 5H7C, 1VW1, 4QKW, 4UXV and 4QKV were chosen as templates to model Cavin1 homology. Similarly, 4UXV, 6H2X, 4QKW, 4QKV and 5YFP were selected as templates for modeling vCavin1 homology. Sequence alignment indicated that the middle segment (46~146) of 4QKW and 4QKV sequences showed high homology with Cavin1 and vCavin1, and that the residues in the HR helix region were correctly matched (Figure 1A). Despite lower homology, the coverage of template 5H7C, 1VW1, 4UXV, 6H2X and 5YFP was higher, which compensated for the low coverage of 4QKW and 4QKV, thereby improving the accuracy of the models.

Next, multi-template homology modeling and clustering was performed using I-TASSER and SPICKER programs, and the structure of the target protein was optimized using Gromacs4.6 kinetic software. The three-dimensional structure of the target protein after modeling and optimization is shown (Figure 1B-C). The schematic diagram of the prediction process and a high accuracy of protein models were shown (Figure S1). Both Cavin1 and vCavin1 have long-chain Helix structures, which enable trimer formation, with some additional loop structures at the end. Models of Cavin1 and vCavin1 were superposed in order to analyze structural differences between them. As shown in Figure 1B, while the structure of middle and end parts of the two proteins were well superimposed, the front-end structure of vCavin1 had switched from Helix to Loop and its orientation had changed, due to fusion of the TAT peptide segment. Potential electrostatic surface views of the monomer and trimer of Cavin1 and vCavin1 are shown. In vCavin1, positively charged regions of the N-terminal structure were all bent outward and the electrostatic surface properties within the telechelic structure were different from those of Cavin1. In Cavin1, the inner sidewalls of the telechelic cavity were mostly negatively charged, while the bottom was positively charged. However, in vCavin1, the inner sidewalls of the telechelic cavity were mostly uncharged or negatively charged.

### Protein docking and molecular dynamic simulation demonstrate unstable binding between vCavin1 and Caveolin1

The structure modeling of Caveolin1 was reasonable and accurate (Figure S2A-B). A hairpin structure of Caveolin1 formed by the transmembrane segments (residues 102-117 and 119-133) is embedded in the plasma membrane (Figure S2C). Due to the presence of a large number of hydrophilic residues at the N-terminus of Caveolin1, the surface of the region containing resides 1-81 was mostly negatively charged, providing a possible site for protein binding (Figure 1D, Figure S2D-E).

Docking between Cavin1/vCavin1 and Caveolin1 was performed using ZDock to predict all possible interaction patterns. The lowest energy conformation was selected for visual analysis via PyMol v1.60. After optimizing the conformation of protein complexes produced by docking, the geometry of the conformation was first analyzed. The spatial parameters are shown (Table 3).

**Figure 2:**
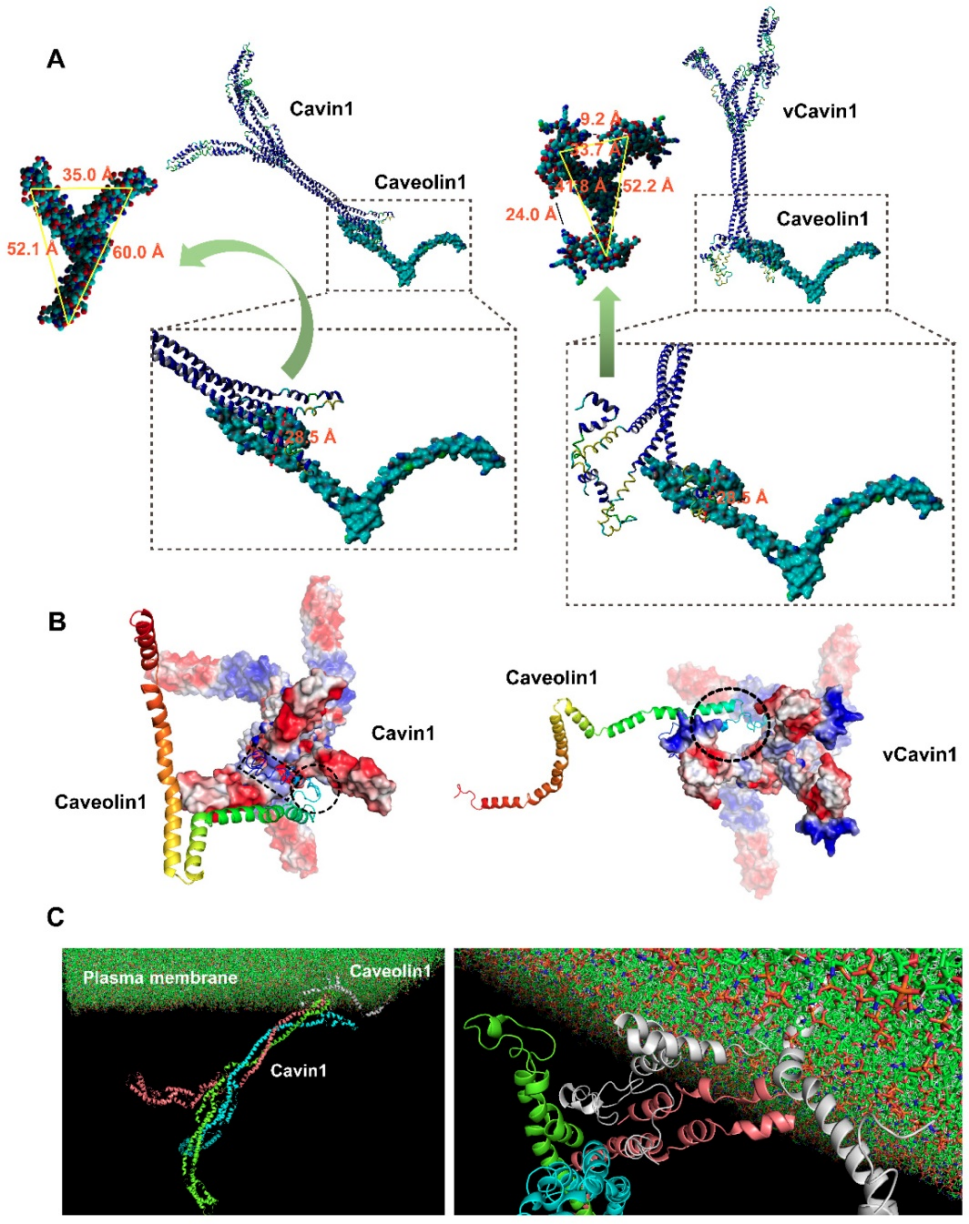
** Protein docking and molecular dynamic simulation of Cavin1 and vCavin1 trimer with Caveolin1.** (A) Binding conformation analysis of Caveolin1 to Cavin1 and vCavin1, respectively. N-terminus of Caveolin1 fits well in the telechelic cavity of Cavin1. However, vCavin1 bound to Caveolin1 only through external structural adsorption. There was no spatial complementarity and restriction between the two proteins. (B) Electrostatic surface view of the potential binding sites of Cavin1/vCavin1 to Caveolin1. The positive and negative charged regions of Cavin1 and Caveolin1 were well matched. Caveolin1 does not match the electrostatic surface of vCavin1. vCavin1 can only bind to the surface of Caveolin1 through the positively charged region of the segment bending outward, thus the stability of the vCavin1-Caveolin1 complex was weakened to some extent. Blue: cationic; white: electroneutral; red: anionic. Rainbow-colored: Caveolin1. (C) Transmembrane Cavin1-Caveolin1 model constructed using molecular dynamic simulation. Cavin1 was anchored on plasma membrane by binding to Caveolin1. Cyan, pink, and green: homotrimer of Cavin1. Grey: Caveolin1.

Cavin1 trimer bound well to the surface of Caveolin1 via Cavin1's front-end telechelic structure, where the depth of this telechelic structure was 25.5 Å (Table 3). The maximum distance between the binding site on Caveolin1 and the cell membrane was 36.0 Å (Table 3). This prevented Cavin1 from colliding with the cell membrane during binding. Moreover, the width of the gap between telechelic jaws was 52.1, 35.0 and 60.0 Å, respectively (Figure 2A), while the maximum size of the Caveolin1 binding site was 28.5 Å (Figure 2A), allowing the N-terminus of Caveolin1 to be well embedded in the Cavin1 telechelic cavity (Figure 2A). The loop structure on the outside area of Caveolin1 formed a bulge and was embedded in the gap between the Cavin1 trimers, which makes the spatial binding between these 2 proteins more stable.

The telechelic cavity of vCavin1 is deeper than that of Cavin1, with a depth of 52.6 Å (Table 3), which is much larger than the maximum distance between Caveolin1 and the plasma membrane, making it easy for vCavin1 to collide with the plasma membrane when docking with Caveolin1. Furthermore, the width of the gaps between the front-end teleports were 9.2 and 24.0 Å (Figure 2A) and thereby smaller than the maximum size of the binding site on Caveolin1. Therefore, it is difficult for Caveolin1 to enter the telechelic cavity of vCavin1 and thus vCavin1 protein can only be bound through external structural adsorption. Spatial complementarity and restriction between vCavin1 and Caveolin1 was absent, and the stability of the formed complex was lower than that of the Caveolin1-Cavin1 protein complex.

Analysis of potential hydrogen bond formation (including hydrogen bond donors, acceptors and bond lengths) between Cavin1 and Caveolin1 is shown (Table 4). The residue in the front-end Helix structure of Cavin1 formed strong hydrogen bonds with the residues in the N-terminus of Caveolin1. Hydrogen bond length between Cavin1 and Caveolin1 was within 1.7 ~ 2.7 Å, and the bond angle ranged from 96.4 to 159.0° (Table 4). These indicated strong hydrogen bond formation. Formation of double hydrogen bonds and strong hydrogen bonds promoted the binding between Cavin1 and Caveolin1, thereby improving the stability of Cavin1-Caveolin1 protein complexes. Results of the analysis of potential hydrogen bond formation between vCavin1 and Caveolin1 are summarized (Table 5). Residues in the front end of vCavin1 formed hydrogen bonds with residues in the Loop structure of the N-terminus of Caveolin1. The number of potential hydrogen bonds between vCavin1 and Caveolin1 was far less than that between Cavin1 and Caveolin1 (Table 5).

Electrostatic surfaces of the sites on Cavin1 and vCavin1 that facilitated binding with Caveolin1 were analyzed. The binding site of Caveolin1 is mostly positively charged, and was very well matched with the negatively charged region on the inner sidewalls of the telechelic cavity of Cavin1 (Figure 2B). However, the corner regions in Helix and Loop structures of Caveolin1 are negatively charged, which match the positively charged region at the bottom of the Cavin1 telechelic cavity, allowing Caveolin1 to be embedded in the bottom and attached to the bottom surface (Figure 2B). Such excellent matching of electrostatic surfaces further improved stability of the Cavin1-Caveolin1 complex. In regard to vCavin1, the positively charged regions of its N-terminal structure are all bent outward (Figure 2B), hindering Caveolin1 from being inserted into its telechelic structure. Therefore, vCavin1 is able to match the negatively charged region of the N-terminus of Caveolin1 (Figure 2B) only through the outwardly bent positively charged region, and this reduces the stability of the Caveolin1-vCavin1 protein complex.

The transmembrane protein complex consists of membrane‑inserted Caveolin1 and cytoplasmic Cavin1 (Figure 2C). Molecular dynamics simulation of this complex was performed using Gromacs 4.6.3 software, and the models were visualized with VMD software. Firstly, after the kinetic simulation process reached equilibrium, six frames from the equilibrium phase were extracted to perform a more detailed comparative analysis. The Caveolin1 and Cavin1 complex did not dissociate at different times in the kinetic process, showing that the binding was stable. The protein complex underwent structural adjustment during the kinetic simulation process, and its stability increased gradually. Secondly, the energy analyzation further confirmed the above observations. The smaller the energy, the more stable the system was. Throughout the simulation process, the total energy of the system gradually decreased and then reached a balance, where there was no obvious fluctuation. In other words, the system became increasingly more stable. Thirdly, we analyzed the centroid distance between Caveolin1 and Cavin1 during the kinetic simulation process. The centroid distance tended to be balanced at 63 Å following system equilibrium, demonstrating the high binding stability of the complex in the solvent system. This provides a theoretical explanation for the high level of adsorption of Caveolin1 by Cavin1, guiding development of the experiments.

### eGFP-Cavin1 in U87 was loaded into EVs in a caveolae-related manner

**Figure 3:**
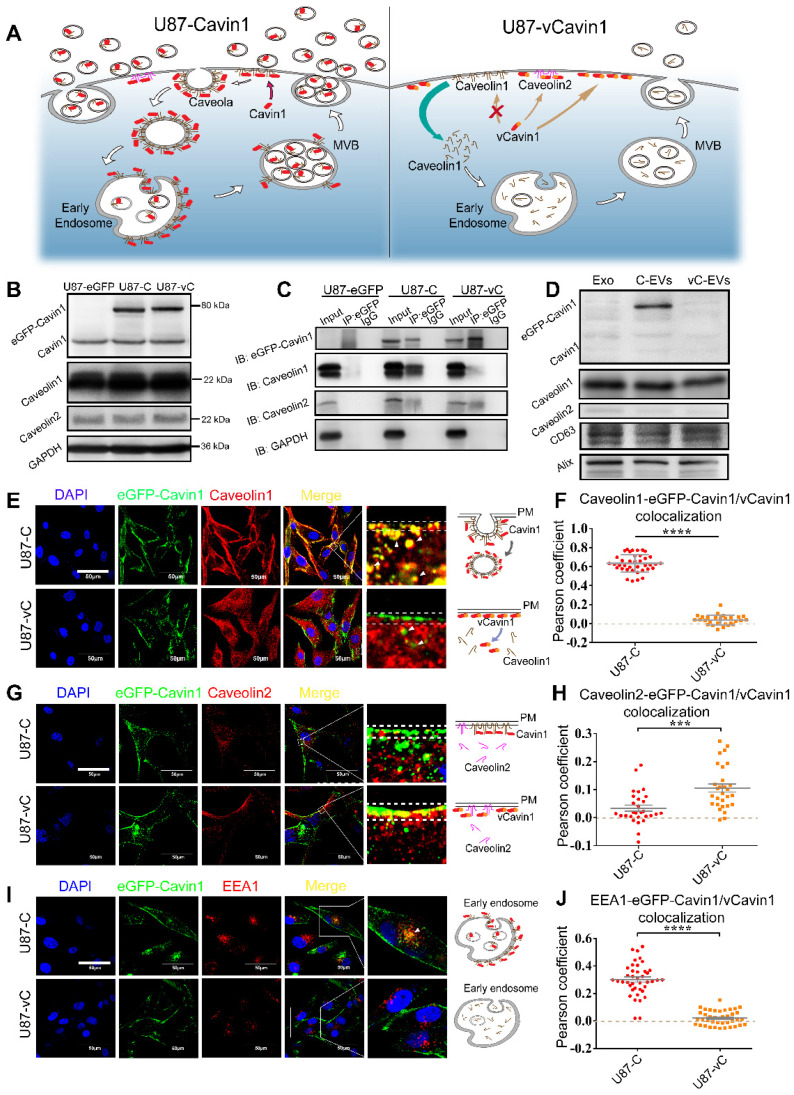
** eGFP-Cavin1 interacts with Caveolin1 and was loaded onto EVs of U87 cells.** (A) Schematic diagram of the proposed mechanism showing how Cavin1 and vCavin1 affect EV production. (B) WB analysis showing an equal expression level of eGFP-Cavin1 and eGFP-vCavin1 in U87 cells. In addition, the levels of endogenous Cavin1, Caveolin1 and Caveolin2 were not altered by eGFP-Cavin1 and eGFP-vCavin1 expression. (C) IP-WB analysis showing an obvious association of eGFP-Cavin1 with Caveolin1 but no binding between eGFP-vCavin1 and Caveolin1 in U87. (D) WB analysis showing the overexpression of eGFP-Cavin1 in EVs, whereas no eGFP-vCavin1 was detected. Besides, eGFP-Cavin1 and eGFP-vCavin1 expression did not affect levels of endogenous Cavin1, Caveolin1, and Caveolin2 in EVs. (E) Confocal images showing an evident co-localization of eGFP-Cavin1 with Caveolin1 but little co-localization of eGFP-vCavin1 with Caveolin1. (F) Colocalization was quantified and expressed as a Pearson coefficient value. Colocalization of Caveolin1 with eGFP-Cavin1 was significantly higher than with eGFP-vCavin1 (p < 0.0001). (G) Confocal images showing co-localization of eGFP-vCavin1 with Caveolin2 but only little co-localization of eGFP-Cavin1 with Caveolin2. (H) The colocalization of Caveolin2 with eGFP-vCavin1 was higher than with eGFP-Cavin1 (p < 0.001). (I) Confocal images showing co-localization of eGFP-Cavin1 with EEA1. (J) Colocalization of EEA1 with eGFP-Cavin1 was significantly higher than with eGFP-vCavin1 (p < 0.0001).

It was hypothesized that overexpressed Cavin1 may localize in caveolae, interact with Caveolin1 and be sorted into EVs in a caveolae-related manner (Figure 3A), whereas, vCavin1 would not interact with Caveolin1, localize in caveolae or be sorted into EVs.

Firstly, eGFP-Cavin1 and eGFP-vCavin1 were expressed in U87, respectively, via lentiviral transfection (U87-C and U87-vC). The expression of exogenous eGFP-Cavin1 and eGFP-vCavin1, as well as endogenous Cavin1, Caveolin1 and Caveolin2 was detected via western blot (WB); (Figure 3B). Full-length blot images of all WB analyses were shown (Figure S4). It was found that eGFP-Cavin1 and eGFP-vCavin1 showed equivalent expression levels and did not alter the expression levels of endogenous Cavin1, Caveolin1, and Caveolin2 in U87 (Figure S5A). Next, immunoprecipitation (IP)-WB analysis was performed to determine the binding of eGFP-Cavin1 or eGFP-vCavin1 to Caveolin1/2. Caveolin1 interacted with eGFP-Cavin1 but did not interact with eGFP-vCavin1 (Figure 3C), which was consistent with protein docking results. In addition, there was a little co-precipitation of Caveolin2 with eGFP-vCavin1 (Figure 3C).

Next, the expression of above proteins in U87-eGFP, U87-C, and U87-vC-derived EVs was analyzed using WB via equal loading of each protein sample. WB analysis revealed that EVs were enriched in overexpressed eGFP-Cavin1 while no eGFP-vCavin1 was detected (Figure 3D), suggesting that sorting of eGFP-vCavin1 into EVs was defective. Importantly, the expression levels of endogenous Cavin1 in the EVs of all 3 groups were low (Figure 3D). In addition, consistent with the results of whole-cell-lysates, eGFP-Cavin1 and eGFP-vCavin1 did not alter the expression level of endogenous Cavin1, Caveolin1 or Caveolin2 in EVs (Figure S5B).

Next, confocal images were analyzed in order to investigate subcellular localization of eGFP-Cavin1 and eGFP-vCavin1. Both eGFP-Cavin1 and eGFP-vCavin1 were localized largely on plasma membrane, with parts of them being localized in the cytoplasm (Figure 3E, G, I). Next, co-localization in whole cells of eGFP-Cavin1 or eGFP-vCavin1 puncta with Caveolin1, Caveolin2 and EEA1, a marker of early endosomes, was quantitatively analyzed (Figure 3E-J). It was observed that eGFP-Cavin1 puncta exhibited a significantly higher degree of co-localization with Caveolin1 than eGFP-vCavin1 (Pearson coefficient, eGFP-Cavin1: 0.63±0.01; eGFP-vCavin1: 0.04±0.01; mean ± SEM, p < 0.0001); (Figure 3F).

Moreover, compared to Caveolin1 located both in the cytoplasm and in the plasma membrane of wild-type U87 cells (Figure S3), Caveolin1 in U87-C seemed to be recruited greatly from the cytoplasm to the plasma membrane (Figure 3E). However, whether in the plasma membrane or in the cytoplasm, eGFP-vCavin1 was dissociated from Caveolin1. And Caveolin1 in U87-vC exhibited a similar subcellular localization with that in wild-type U87 cells (Figure 3E, Figure S3). It suggests that eGFP-Cavin1 can bind extensively to Caveolin1 and co-localizes with Caveolin1 on plasma membrane, whereas eGFP-vCavin1 cannot.

Although the co-localization levels of Caveolin2 with either eGFP-Cavin1 or eGFP-vCavin1 were low, eGFP-vCavin1 exhibited a higher level of co-localization with Caveolin2 than eGFP-Cavin1 (Pearson coefficient, eGFP-Cavin1: 0.03±0.01; eGFP-vCavin1: 0.11±0.01; mean ± SEM, p < 0.001); (Figure 3H). Additionally, there was more co-localization between eGFP-Cavin1 and EEA1 than between eGFP-vCavin1 and EEA1 (Pearson coefficient, eGFP-Cavin1: 0.30±0.02; eGFP-vCavin1: 0.02±0.01; mean ± SEM, p < 0.0001); (Figure 3J). This indicated that eGFP-Cavin1 was able to enter early endosomes whereas eGFP-vCavin1 could not.

Above evidence indicated that interaction with Caveolin1 was essential for Cavin1 to enter EVs. Thus, a caveolae-related pathway may play a key role in the recruitment of Cavin1 to EVs.

### eGFP-Cavin1 increased EV secretion with no effect on morphology and average size

**Figure 4:**
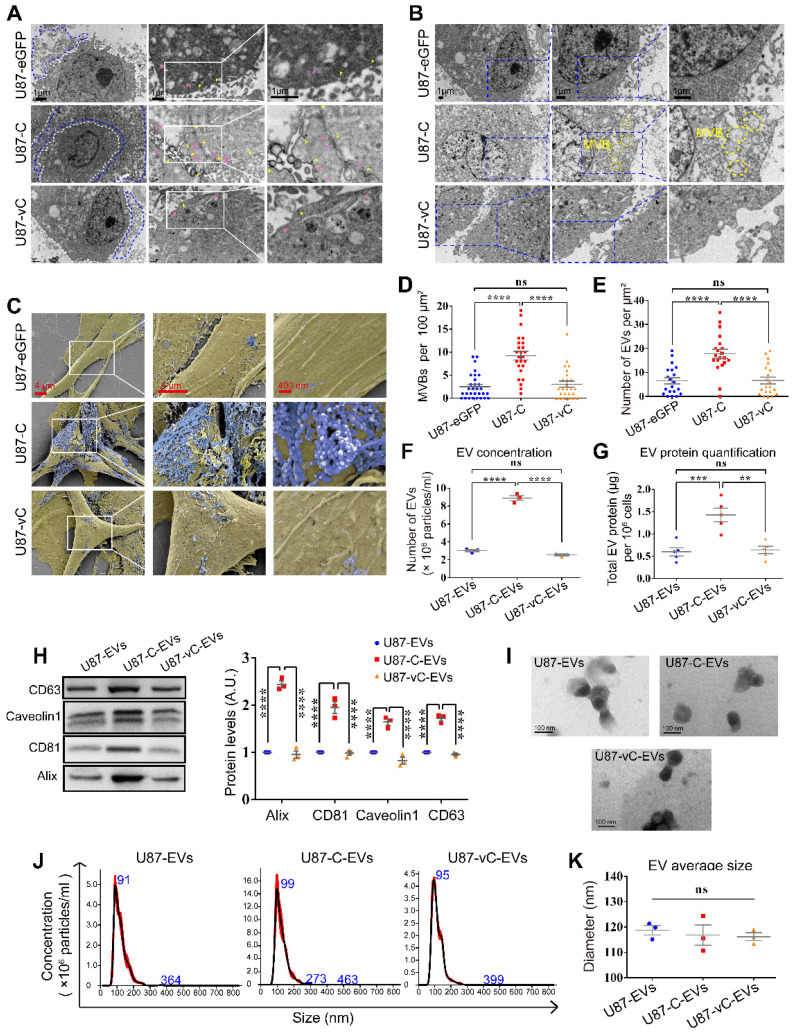
** eGFP-Cavin1 expression enhanced U87-EV production.** (A) TEM images showing increased EVs, caveolae and endocytic vesicles in Cavin1 overexpressing U87 cells. The area surrounded by the white dotted line is the cell body, while the area surrounded by the blue dotted line is the extracellular space rich in EVs. Yellow arrowheads represent caveolae and pink arrowheads represent endocytic vesicles. (B) TEM images showing increased MVBs. The areas surrounded by the yellow dotted line are MVBs. (C) SEM images showing abundant EVs attached to the extracellular surface of U87-C. Cell bodies are rendered in yellow, and the EVs attached to the outer surface of the cells are rendered in blue. (D-E) The number of MVBs (per 100 μm^2^) (D), and the number of EVs (per μm²) of U87-eGFP, U87-C, and U87-vC (E). Data are expressed as the mean ± SEM. (F) EV concentration was expressed as the number of EVs (×10^8^ particles/mL). The concentration of U87-C-EVs was significantly higher than that of U87-EVs and U87-vC-EVs (p < 0.0001; p < 0.0001). (G) EV protein quantification was performed by measuring the total EV protein (μg) per 10^6^ cells. The protein concentration of U87-C-EVs was higher than that of U87-EVs and U87-vC-EVs (p < 0.001; p < 0.01). (H) EVs were isolated from an equal volume of cell culture supernatant and the expression of several proteins in EVs was analyzed. The levels of CD63, Alix, CD81, and Caveolin1 in U87-C-EVs were elevated as compared with that in U87-EVs and U87-vC-EVs (p < 0.0001; p < 0.0001). (I) Representative TEM images showing the morphologies of U87-EVs, U87-C-EVs and U87-vC-EVs. (J) NTA analysis showing a similar particle size distribution of U87-EVs, U87-C-EVs and U87-vC-EVs. (K) No significant difference between the average diameters of U87-EVs, U87-C-EVs and U87-vC-EVs (p > 0.05).

To investigate the effects Cavin1 exerts on EV secretion, transmission electron microscopy (TEM) and scanning electron microscopy (SEM) were applied to characterize intracellular caveolae, multivesicular bodies and extracellular small vesicles (Figure 4A-C). Compared with the control (U87-eGFP) and vCavin1 (U87-vC) groups, more caveolae structures (yellow arrows) and endocytic vesicles (pink arrows) were observed in eGFP-Cavin1-overexpressing U87 cells (U87-C); (Figure 4A). In addition, MVBs were found to be increased in U87-C as compared with U87-eGFP and U87-vC (Number of MVBs per 100 μm^2^, U87-eGFP: 2.50±0.50; U87-C: 9.24±0.92; U87-vC: 3.04±0.67; mean ± SEM, p < 0.0001); (Figure 4B, D). MVBs, characterized by the presence of intraluminal vesicles (ILVs), can fuse with the plasma membrane leading to the secretion of small EVs/exosomes to the extracellular environment. Increased MVBs may indicate the active production of small EVs/exosomes in U87-C. Therefore, SEM was used to observe secreted EVs in the extracellular space (Figure 4C). As expected, there were more EVs on the outer surface of the cell membrane of U87-C than on U87-eGFP and U87-vC (Number of EVs per μm^2^, U87-eGFP: 6.50±1.32; U87-C: 17.75±1.86; U87-vC: 6.60±1.41; mean ± SEM, p < 0.0001); (Figure 4E). In order to conduct a quantitative comparison of EV secretion by the 3 groups during a given period of time, EVs derived from an equal number of cells in each of the 3 groups were analyzed for EV concentration and protein content. The concentration of U87-C-EVs was significantly higher than U87-EVs and U87-vC-EVs (Number of EVs [10^8^ particles/mL], U87-EVs: 3.02±0.12; U87-C-EVs: 8.88±0.28; U87-vC-EVs: 2.56±0.11; mean ± SEM, p < 0.0001) (Figure 4F). The protein content of U87-C-EVs was higher compared to that of U87-EVs and U87-vC-EVs (Total EV protein [μg] per 10^6^ cells, U87-EVs: 0.60±0.09; U87-C-EVs: 1.43±0.15; U87-vC-EVs: 0.64±0.08; mean ± SEM, p < 0.001 and p < 0.01); (Figure 4G). The expression of several proteins including Alix, CD81, Caveolin1 and CD63, which exhibited relatively stable expression levels in U87-derived EVs, were analyzed in the 3 groups (Figure 4H). Consistent with the results of EV quantification, the expression levels of Alix, CD81, Caveolin1 and CD63 were elevated in U87-C-EVs compared to those in U87-EVs and U87-vC-EVs (Figure 4H).

Next, the morphology and size of EVs in the 3 groups was investigated. The EVs displayed cup-shaped structures under a transmission electron microscope (Figure 4I). Furthermore, particle size analysis showed that there was no difference among the average EV sizes of the 3 groups (Diameter [nm], U87-EVs: 118.8±1.84; U87-C-EVs: 116.9±4.01; U87-vC-EVs: 116.2±1.59; mean ± SEM, p > 0.05); (Figure 4J-K).

These results suggested that eGFP-Cavin1 in U87 cells enhanced EV secretion without altering average particle size.

### eGFP-Cavin1 in U87 cells increased EV uptake by recipient LN229 cells and promoted the proliferation of LN229

Having verified that Cavin1 overexpression enhanced U87-EV secretion, we next investigated whether Cavin1 expression affected EV uptake by recipient cells.

**Figure 5:**
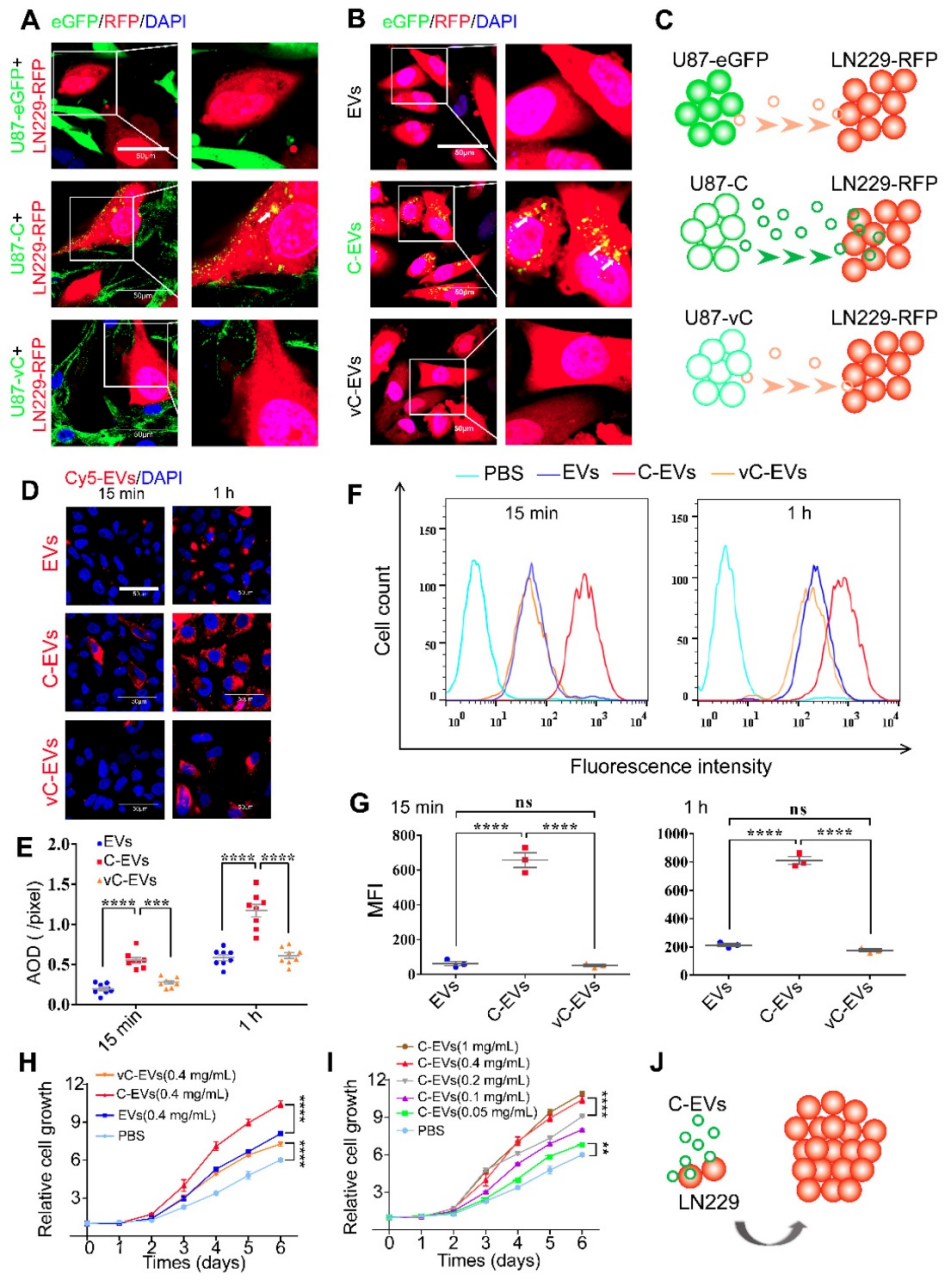
** eGFP-Cavin1 expression in U87 increased EV uptake and the proliferation of recipient cells LN229.** (A) Confocal images showing the co-culture of LN229-RFP cells with an equal number of U87-eGFP, U87-C, and U87-vC cells, respectively; eGFP-Cavin1 was transferred from U87-C to LN229-RFP cells (the white arrow). (B) Confocal images showing the LN229-RFP cells incubated with an equal concentration of U87-EVs, U87-C-EVs, and U87-vC-EVs, respectively; eGFP-Cavin1 was transferred via EVs to LN229-RFP cells (white arrows). (C) A schematic diagram describing the transfer of eGFP-Cavin1 from U87 to LN229 via EVs, whereas eGFP and eGFP-vCavin1 were not transferred via EVs. (D) Confocal images showing LN229 cells incubated with an equal concentration of Cy5-labeled U87-EVs, U87-C-EVs and U87-vC-EVs for 15 min and 1 h, respectively. (E) Quantitation of the average optical density (AOD) of Cy5 in LN229 cells. At 15 min and 1 h post incubation, cells incubated with U87-C-EVs showed an increased AOD of Cy5 fluorescence. (F) Flow cytometry analysis of Cy5 fluorescence in LN229 cells. (G) Geo Mean fluorescence intensity (MFI) of Cy5 in LN229 cells analyzed through flow cytometry. At 15 min and 1 h post incubation, an increased MFI in cells incubated with U87-C-EVs (15 min: C-EVs *vs* EVs, p < 0.0001; C-EVs *vs* vC-EVs, p < 0.0001. 1h: C-EVs *vs* EVs, p < 0.0001; C-EVs *vs* vC-EVs, p < 0.0001). (H) CCK8 assay showing an increased cell growth of LN229 treated with U87-C-EVs than those treated with U87-EVs or U87-vC-EVs from day 3 (At day 6, C-EVs *vs* EVs, p < 0.0001; C-EVs *vs* vC-EVs, p < 0.0001). (I) The relative cell growth of LN229 treated with a series of increasing concentrations of U87-C-EVs. In a range of 0.05-0.4 mg/mL, as the concentration of U87-C-EVs increased, the proliferation of LN229 exhibited an increasing trend (At day 6, 0.05 mg/mL *vs* PBS, p < 0.01; 0.1 mg/mL *vs* 0.05 mg/mL, p < 0.0001; 0.4 mg/mL *vs* 0.2 mg/mL, p < 0.0001; 1 mg/mL *vs* 0.4 mg/mL, p > 0.05). (J) A schematic diagram describing efficient internalization of U87-C-EVs by LN229 efficiently and increased LN229 proliferation.

First, LN229 glioma cells, used as recipient cells, were transfected with lentivirus-red fluorescence protein (LV-RFP). LN229-RFP were co-cultured for 96 h with an equal number of U87-eGFP, U87-C and U87-vC, respectively, and observed via a confocal microscope (Figure 5A). Confocal images showed eGFP-Cavin1 could be detected in LN229-RFP cells (white arrow; Figure 5A) whereas eGFP and eGFP-vCavin1 could not be detected. This suggested that eGFP-Cavin1 could be transferred among glioma cells. To further ascertain whether the delivery of eGFP-Cavin1 was mediated by EVs, LN229-RFP were treated with 0.5 mg/mL of EVs derived from U87-eGFP, U87-C and U87-vC, respectively, and incubated for 6 h. Similarly, only eGFP-Cavin1 was detected in LN229-RFP (Figure 5B), suggesting that the transport of eGFP-Cavin1 among glioma cells was at least in part mediated by EVs (Figure 5C). Moreover, eGFP-vCavin1 was not detected in recipient cells, indicating that normal interaction with Caveolin1 plays an important role not only in the sorting of Cavin1 into EVs but also in transporting Cavin1 among glioma cells.

In order to quantitatively assess the internalization of U87-EVs, U87-C-EVs and U87-vC-EVs into LN229 cells, respectively, Cy5-labeled EVs (0.5 mg/mL) were added to the culture medium of LN229. LN229 cells were collected after 15 min and 1 h, and Cy5 fluorescence intensity was analyzed using confocal microscopy and flow cytometry. U87-C-EVs internalized by LN229 exhibited a higher level of Cy5 intensity than that of U87-EVs and U87-vC-EVs both at 15 min and at 1h (Average Optical Density, AOD [pixels], 15 min, U87-EVs: 0.19±0.02, U87-C-EVs: 0.55±0.04, U87-vC-EVs: 0.28±0.02; 1 h, U87-EVs: 0.59±0.04, U87-C-EVs: 1.17±0.08, U87-vC-EVs: 0.61±0.04; mean ± SEM); (Figure 5D-E). Similar results were verified using flow cytometry (GeoMean Fluorescence Intensity, MFI, 15 min, U87-EVs: 62.00±12.66, U87-C-EVs: 656.7±41.57, U87-vC-EVs: 50.87±6.96; 1 h, U87-EVs: 213.3±10.97, U87-C-EVs: 810.0±26.76, U87-vC-EVs: 175.0±10.44; mean ± SEM) (Figure 5F-G). Therefore, our results showed that Cavin1 overexpression in U87 cells increased EV uptake by recipient cells, LN229. Additionally, we found that U87-C-EVs increased the proliferation rate of LN229 cells in a dose-dependent manner. LN229 cells treated daily with 0.4 mg/mL of U87-C-EVs showed significantly increased proliferation than those treated with U87-EVs and U87-vC-EVs from day 3 (Figure 5H). However, there were no significant differences between the proliferation of U87-EV and U87-vC-EV treated LN229 cells (Figure 5H). Furthermore, as concentration was increased within the range of 0.05~0.4 mg/mL, the proliferation-promoting effect of U87-C-EVs tended to be elevated (Figure 5I).

### eGFP-Cavin1 enhanced EV-mediated transport of eGFP-Cavin1 and recipient cell proliferation in orthotopic xenograft glioma mice

**Figure 6:**
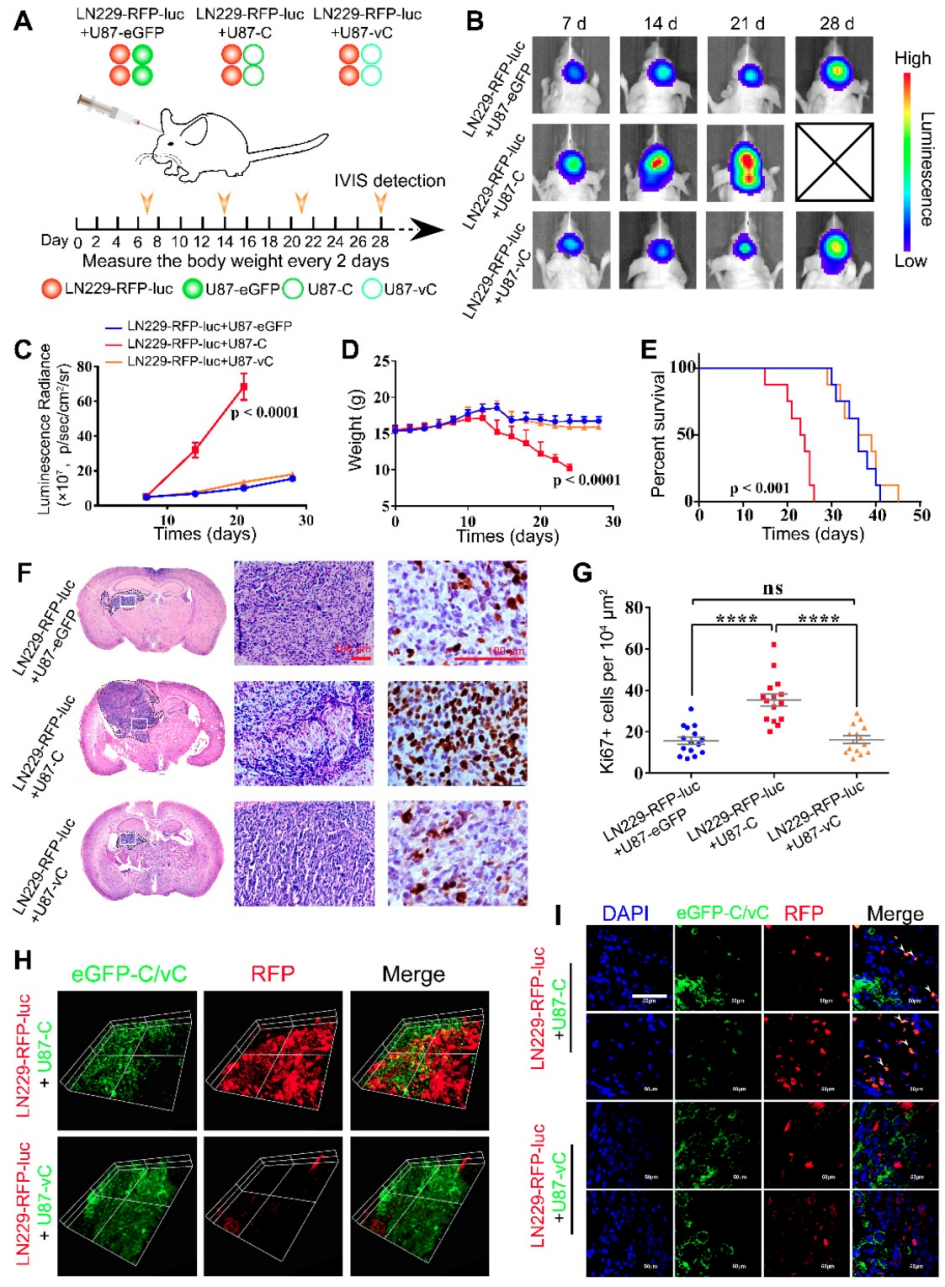
** eGFP-Cavin1 was transferred via EVs to recipient LN229 cells and increased LN229 proliferation in orthotopic xenograft glioma mice.** (A) Schematic illustration of experimental grouping and process of the mixed glioma xenograft model. U87-eGFP, U87-C, and U87-vC were respectively mixed with an equal number of LN229-RFP-luc and implanted intracranially in nude mice. IVIS detection was performed at day 7, 14, 21, and 28 post-implantation (n = 8), and brains were dissociated at day 21 for histological analysis and confocal imaging. (B) *In vivo* bioluminescence imaging showing a higher signal intensity in mice implanted with LN229-RFP-luc+U87-C. (C) Analysis of the bioluminescence intensity suggesting a rapidly increasing growth of LN229-RFP-luc which were mixed with U87-C from day 7. (D) Weight analysis indicating a faster weight loss in mice implanted with LN229-RFP-luc+U87-C from day 12 (n = 8). (E) Kaplan-Meier survival curves showing the percent survival of mice implanted with LN229-RFP-luc+U87-eGFP, LN229-RFP-luc+U87-C, and LN229-RFP-luc+U87-vC, respectively (n = 8, p < 0.001; log-rank test). (F) H&E and Ki67 staining of mouse cerebrum with tumor which was harvested at day 21 post implantation (n = 5). H&E staining showing a more heterogeneous composition in the LN229-RFP-luc+U87-C tumor. Scale bar, 100 µm. (G) IHC for Ki67 showing an increased number of Ki67-positive cells in the LN229-RFP-luc+U87-C tumor (mean±SEM, p < 0.0001; p < 0.0001). (H) 3D images generated from the Z-stacks with a slice-distance of 1.6 μm, showing that the number of LN229-RFP-luc cells co-implanted with U87-C were increased and eGFP-Cavin1 was transferred to LN229-RFP-luc (n = 4). (I) Confocal images clearly showing the transfer of eGFP-Cavin1 to LN229-RFP-luc cells (white arrowheads) whereas no transfer of eGFP-vCavin1 was detected in LN229-RFP-luc cells (n = 4).

To gain insight into the role of Cavin1 in inter-glioma cell communication *in vivo*, we established an intracranial mixed-glioma model. LN229 cells were transduced with lentiviruses expressing RFP and firefly luciferase, and mixed with an equal number of U87-eGFP, U87-C, or U87-vC, respectively (LN229-RFP-luc+U87-eGFP, LN229-RFP-luc+U87-C, and LN229-RFP-luc+U87-vC). Then 5×10^5^ of mixed cells were implanted into the intracranium of nude mice (Figure 6A). At day 7, 14, 21, and 28 post implantation, tumor growth was monitored via bioluminescence imaging (Figure 6B). The luminescence radiance reflected LN229 proliferation in vivo. LN229-RFP-luc+U87-C implanted mice exhibited significantly higher luminescence radiance than LN229-RFP-luc+U87-eGFP and LN229-RFP-luc+U87-vC implanted mice from day 14 (p < 0.0001; Figure 6B-C), which suggests an enhanced growth-promoting effect of U87-C on LN229. Mice were monitored daily and weighed every other day. The body weight of LN229-RFP-luc+U87-C implanted mice decreased faster than that of LN229-RFP-luc+U87-eGFP and LN229-RFP-luc+U87-vC implanted mice from day 14 post implantation (p < 0.0001); (Figure 6D). Furthermore, Kaplan-Meier survival analysis indicated that LN229-RFP-luc+U87-C implantation was significantly associated with poorer overall survival compared with LN229-RFP-luc+U87-eGFP and LN229-RFP-luc+U87-vC (P < 0.0001); (Figure 6E).

For histologic analysis, 5 mice from each group were sacrificed on day 21, and brains were excised and processed into paraffin embedded sections. Hematoxylin and eosin (H&E) staining showed that LN229-RFP-luc+U87-C exhibited a higher degree of heterogeneity and a more aggressive growth potential (Figure 6F). In addition, Ki67 staining confirmed a more active proliferation of LN229-RFP-luc+U87-C as compared with LN229-RFP-luc+U87-eGFP and LN229-RFP-luc+U87-vC (Ki67^+^ cells per 10^4^ μm^2^, LN229-RFP-luc+U87-eGFP: 15.73±1.77, LN229-RFP-luc+U87-C: 35.40±2.91, LN229-RFP-luc+U87-vC: 16.27±1.74; mean ± SEM; p < 0.0001 and p < 0.0001); (Figure 6G).

Next, in order to determine eGFP-Cavin1/vCavin1 transport *in vivo*, mouse brains were harvested on day 21 and processed into frozen sections with a thickness of 8 μm and 80 μm. The 80 μm-thick sections were used to perform Z-axis imaging. As shown in Figure 6H, whereas LN229-RFP-luc mixed with U87-C seemed to be more aggressive than LN229-RFP-luc mixed with U87-vC, eGFP-Cavin1 (green) was transferred from U87-C to LN229-RFP-luc. High magnification (×1000) confocal images further verified the transfer of eGFP-Cavin1 (white arrowheads, Figure 6I), and the absence of eGFP-vCavin1 inside LN229-RFP-luc (Figure 6I).

In view of the difference in EV secretion and cargo between U87-C and U87-vC, we speculated that eGFP-Cavin1 transport between glioma cells *in vivo* was mainly mediated by EVs. Thus, the *in vivo* assay revealed that eGFP-Cavin1 overexpression enhanced EV-mediated transport of eGFP-Cavin1 and recipient cell proliferation.

### EVs expressing eGFP-Cavin1 exhibited a homing tendency towards intracranial glioma when systematically administered

**Figure 7:**
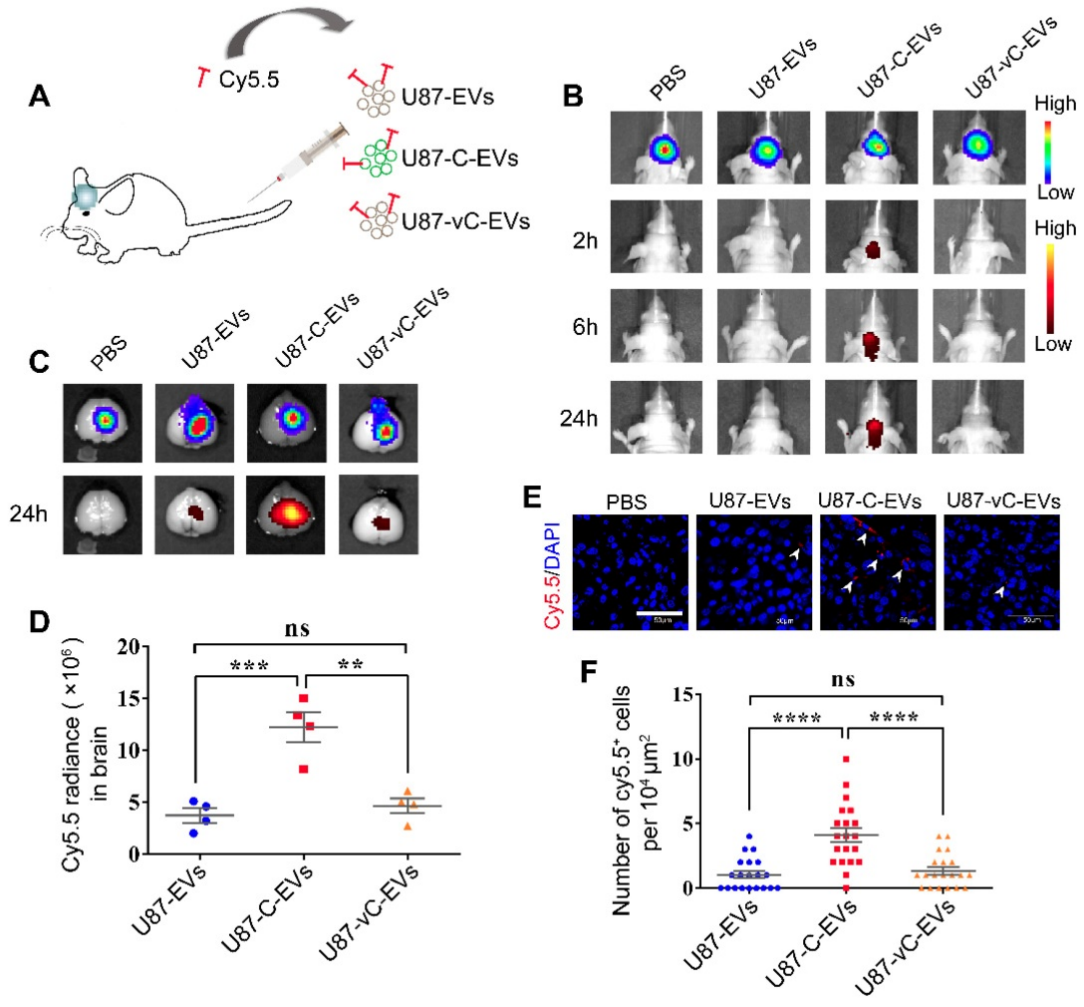
** Systematically applied U87-C-EVs exhibited a homing property towards orthotopic glioma.** (A) Schematic representative of the administering of an equal protein amount of Cy5.5-labeled U87-EVs, U87-C-EVs, and U87-vC-EVs to glioma-bearing nude mice via the tail vein. (B) In vivo bioluminescence imaging performed at 21 d post-implantation of glioma cells and *in vivo* Cy5.5 fluorescence imaging carried out at 2, 6 and 24 h post-EV injection (n = 9). U87-C-EVs accumulated significantly more in mouse brains at 2, 6, and 24 h post-injection. (C) Ex vivo bioluminescence and Cy5.5 fluorescence imaging of mouse brains harvested following the last *in vivo* imaging (24 h post-injection, n = 4). EVs in the brain accumulated mostly inside glioma. (D) Quantification of Cy5.5 fluorescence intensity in brain showing an increased accumulation of U87-C-EVs in brain than U87-EVs and U87-vC-EVs (p < 0.001; p < 0.01). (E) Confocal images of glioma tissues harvested at 24 h post-injection of PBS, Cy5.5 labeled U87-EVs, U87-C-EVs and U87-vC-EVs (n = 5). Cy5.5 positive glioma cells indicated that the Cy5.5-labeled EVs were internalized by these cells (white arrowheads). (F) Quantification of the number of Cy5.5 positive cells per 10^4^ μm in glioma tissue. U87-C-EVs showed more internalization in glioma cells than U87-EVs and U87-vC-EVs (p < 0.0001; p < 0.0001).

To determine whether Cavin1 expression affects the distribution of systematically applied EVs in glioma, equivalent U87-EVs, U87-C-EVs, and U87-vC-EVs were labeled with Cy5.5 dye and intravenously injected via the tail vein (Figure 7A). At 2, 6 and 24 h post injection, fluorescent bio-imaging was performed. U87-C-EVs showed accumulation in the brain as early as 2 h post-injection, and the fluorescent signals in the brain persisted for > 24 h (Figure 7B). However, Cy5.5 signals were almost undetectable in the brains of U87-EV and U87-vC-EV injected mice (Figure 7B). Following *in vivo* imaging at 24 h post-injection, mice were sacrificed and brains were dissected for imaging. Fluorescence intensity of the tumor region was significantly higher in U87-C-EV injected mice as compared to that in U87-EV and U87-vC-EV injected mice (U87-C-EVs *vs* U87-EVs, p < 0.001; U87-C-EVs *vs* U87-vC-EVs; p < 0.01); (Figure 7C-D). However, there was no significant difference in the fluorescence intensity in tumors between U87-EV and U87-vC-EV injected mice (p > 0.05); (Figure 7D). To visualize EVs internalized by glioma cells *in vivo*, mice were sacrificed at 24 h post-injection and transcardially perfused with cold PBS to remove EVs circulating in blood. The tumors were made into frozen sections and analyzed via confocal microscopy.

There were more Cy5.5 positive glioma cells in U87-C-EV injected mice than in U87-EV and U87-vC-EV injected mice (U87-C-EVs *vs* U87-EVs, p < 0.0001; U87-C-EVs *vs* U87-vC-EVs, p < 0.0001); (Figure 7E-F). The results were consistent with those from *in vivo* imaging, thereby illustrating the homing properties of U87-EVs overexpressing Cavin1.

### Cavin1-overexpressing murine glioma cells GL261 secreted EVs leading to recruitment and activation of microglia

Recent studies have suggested that apart from communication between glioma cells, communication with and manipulation of other cells such as microglia and astrocytes in the brain is also crucial for the formation of gliomal microenvironment and tumor progression 29-31. Hence, we investigated whether Cavin1 expression affected EV-mediated communication between glioma cells and microglia.

**Figure 8:**
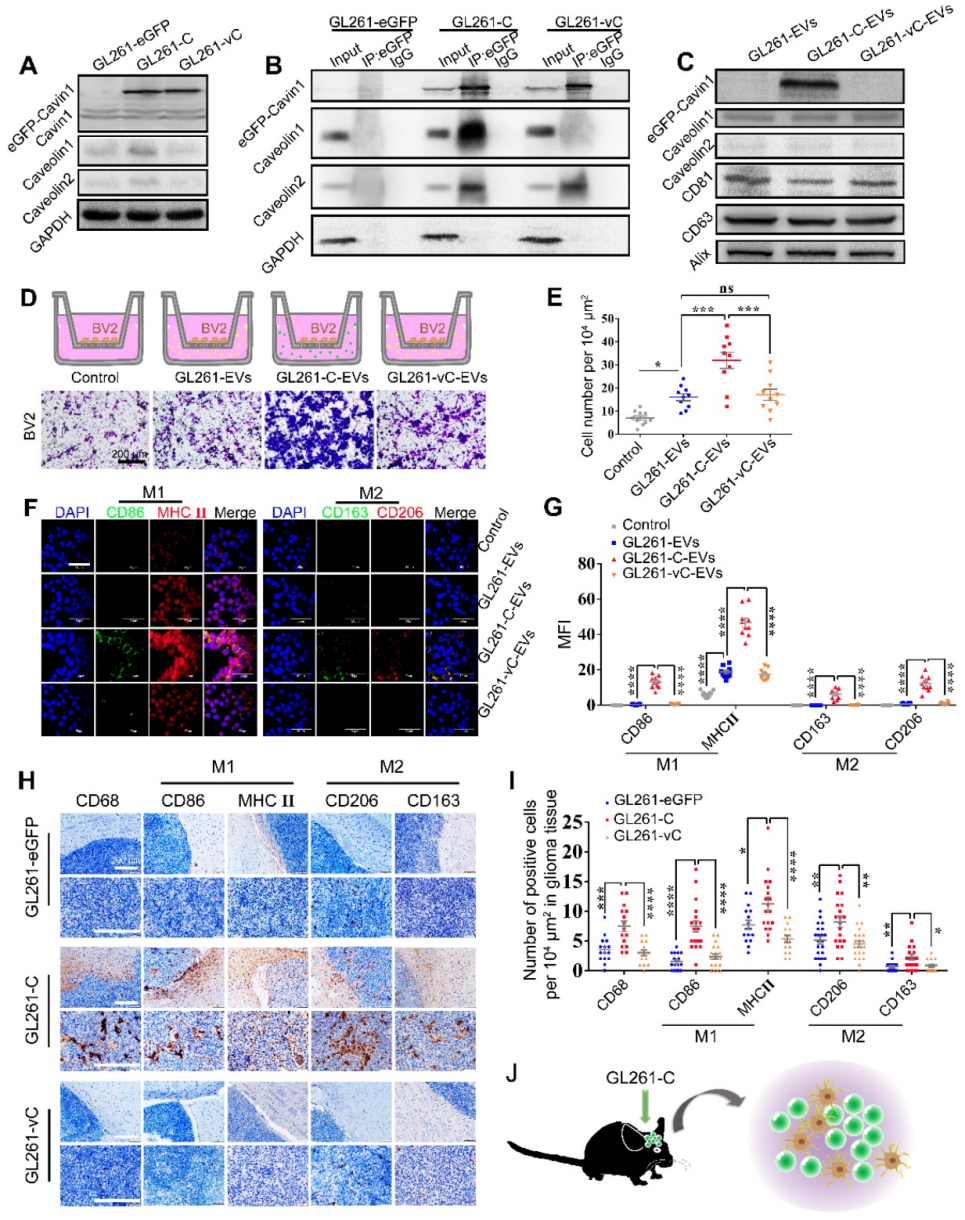
** eGFP-Cavin1 overexpressing murine glioma cells GL261 secreted EVs leading to recruitment and activation of microglia.** (A) WB analysis of the expression of eGFP-Cavin1, endogenous Cavin1, Caveolin1, and Caveolin2 in GL261-eGFP, GL261-C, and GL261-vC cells; eGFP-Cavin1 or eGFP-vCavin1 expression did not alter the expression level of endogenous Cavin1. However, Caveolin1 and Caveolin2 levels increased in cells expressing eGFP-Cavin1. (B) IP-WB analysis showing an obvious association between eGFP-Cavin1 and Caveolin1 but no binding between eGFP-vCavin1 and Caveolin1 in GL261. (C) WB analysis of the expression of eGFP-Cavin1, Caveolin1, Caveolin2, CD63, CD81, and Alix in GL261-EVs, GL261-C-EVs, and GL261-vC-EVs; eGFP-Cavin1 showed a high expression level whereas eGFP-vCavin1 was not detected in EVs. In addition, Caveolin1 and Caveolin2 levels were not affected by eGFP-Cavin1 and eGFP-vCavin1 expression. (D) Representative images of migrated BV2 cells induced by an equal concentration of GL261-EVs, GL261-C-EVs or GL261-vC-EVs in the transwell migration assay. Scale bar, 200 μm. (E) Quantification of the number of migrated BV2 cells. GL261-C-EVs induced an increase in the number of migrated BV2 cells as compared with GL261-EVs and GL261-vC-EVs (p < 0.001; p < 0.001). Compared with the Control group, GL261-EVs and GL261-vC-EVs both increased the migrated cell number (p < 0.05; p < 0.05). (F) Confocal immunofluorescence images of BV2 cells treated for 48 h with an equal concentration of GL261-EVs, GL261-C-EVs and GL261-vC-EVs, respectively, showing the expression of M1 markers (CD86; MHCⅡ) and M2 markers (CD206; CD163) in BV2 cells. (G) Quantification of the GeoMean Fluorescence Intensity (MFI) of CD86, MHC Ⅱ, CD206 and CD163. The MFI of M1 markers (CD86; MHCⅡ) and M2 markers (CD206; CD163) increased in BV2 cells treated with GL261-C-EVs. CD86: GL261-C-EVs *vs* GL261-EVs, p < 0.0001; GL261-C-EVs *vs* GL261-vC-EVs, p < 0.0001. MHCⅡ: GL261-C-EVs *vs* GL261-EVs, p < 0.0001; GL261-C-EVs *vs* GL261-vC-EVs, p < 0.0001. CD163: GL261-C-EVs *vs* GL261-EVs, p < 0.0001; GL261-C-EVs *vs* GL261-vC-EVs, p < 0.0001. CD206: GL261-C-EVs *vs* GL261-EVs, p < 0.0001; GL261-C-EVs *vs* GL261-vC-EVs, p < 0.0001. (H) IHC images showing the expression of CD68, CD86, MHCⅡ, CD206, and CD163 in the glioma tissue of C57BL/6 mice which were implanted with GL261-eGFP, GL261-C, and GL261-vC cells, respectively (n = 6). (I) Quantification of the number of CD68, CD86, MHCⅡ, CD206, and CD163 positive cells in glioma tissue. CD68, CD86, MHCⅡ, CD206, and CD163 positive cells were increased in GL261-C glioma as compared with GL261-eGFP and GL261-vC gliomas. CD68: GL261-C *vs* GL261-eGFP, p < 0.001; GL261-C *vs* GL261-vC, p < 0.0001. CD86: GL261-C *vs* GL261-eGFP, p < 0.0001; GL261-C *vs* GL261-vC, p < 0.0001. MHCⅡ: GL261-C *vs* GL261-eGFP, p < 0.05; GL261-C *vs* GL261-vC, p < 0.0001. CD206: GL261-C *vs* GL261-eGFP, p < 0.01; GL261-C *vs* GL261-vC, p < 0.01. CD163: GL261-C *vs* GL261-eGFP, p < 0.01; GL261-C *vs* GL261-vC, p < 0.05. (J) Schematic illustration of increased infiltration of activated microglia/macrophages in GL261-C glioma.

Murine glioma cell line GL261 were transfected with lentiviruses expressing eGFP, eGFP-Cavin1 and eGFP-vCavin1, respectively (GL261-eGFP, GL261-C and GL261-vC). WB analysis showed that the expression levels of eGFP-Cavin1 and eGFP-vCavin1 comparable (p > 0.05); (Figure 8A; Figure S5C), but both Caveolin1 and Caveolin2 levels in GL261-C were increased as compared with those in GL261-eGFP and GL261-vC (Caveolin1, GL261-C *vs* GL261-eGFP , p < 0.01; GL261-C *vs* GL261- vC , p < 0.01; Caveolin2, GL261-C *vs* GL261-eGFP, p < 0.01; GL261-C *vs* GL261- vC , p < 0.01); (Figure 8A; Figure S5C). In addition, the endogenous Cavin1 levels of the 3 groups were low and there were no significant differences between them (p > 0.05); (Figure 8A; Figure S5C). Next, we verified the interaction of eGFP-Cavin1 with Caveolin1 in GL261 cells via IP-WB analysis and no interaction between eGFP-vCavin1 and Caveolin1 was detected (Figure 8B). Subsequently, EVs derived from GL261-eGFP, GL261-C, and GL261-vC (GL261-EVs, GL261-C-EVs and GL261-vC-EVs) were characterized and quantified (Figure S6B-F). The results indicated that Cavin1 overexpression significantly increased EV secretion by GL261 cells (p < 0.001); (Figure S6D-E). However, vCavin1 expression in GL261 did not alter the production of EVs (p > 0.05); (Figure S6D-E). In addition, neither Cavin1 nor vCavin1 altered the average size of EVs (p > 0.05); (Figure S6F).

Importantly, eGFP-Cavin1 was detected in EVs whereas eGFP-vCavin1 was not (Figure 8C; Figure S5D). The results for GL261 and U87 were consistent, emphasizing the importance of Cavin1-Caveolin1 interaction in the production and protein recruitment (Cavin1) of EVs.

Next, we focused on the effects exerted by GL261-C-EVs on microglia cells, BV2. When administered with an equal concentration in the lower chamber, GL261-C-EVs showed a significantly larger chemo-attraction to BV2 cells in the upper chamber as compared with GL261-EVs and GL261-vC-EVs (GL261-C-EVs *vs* GL261-EVs, p < 0.001; GL261-C-EVs *vs* GL261-vC-EVs, p < 0.001) (Figure 8D-E). There was no significant difference in the number of migrating BV2 between GL261-EVs and GL261-vC-EVs groups (p > 0.05); (Figure 8D-E). These results implied that Cavin1 overexpressing EVs enhanced BV2 recruitment.

Then we investigated whether Cavin1 expression in EVs affected the activation of microglia. Following treatment with 0.6 mg/mL each of GL261-EVs, GL261-C-EVs and GL261-C-EVs for 48 h, BV2 were harvested and subjected to an immunofluorescence assay. The expression levels of several biomarkers of M1 and M2 microglia were elevated in BV2 treated with GL261-C-EVs (p < 0.0001); (Figure 8F-G), suggesting that GL261-C-EVs exerted an overall activating effect on BV2. To further confirm this effect *in vivo*, GL261 cells expressing eGFP, eGFP-Cavin1 and eGFP-vCavin1, respectively, were intracranially injected into C57BL/6 mice (GL261-eGFP, GL261-C, and GL261-vC). On the 35 d post-injection, mice were euthanized and their brains were processed into paraffin-embedded sections for immunohistochemistry analysis. The levels of the marker for the general activated microglia, CD68, markers for M1 microglia (CD86 and MHC Ⅱ) and markers for M2 microglia (CD206 and CD163) were all increased in GL261-C glioma tissue than in GL261-eGFP and GL261-vC glioma tissues (Figure 8H-I). Therefore, Cavin1 overexpressing GL261 enhanced the infiltration of activated microglia inside the tumor (Figure 8J).

## Discussion

This study revealed a hitherto unknown role of Cavin1 in EV-mediated communication not only between glioma cells, but also between glioma cells and microglia. Moreover, we demonstrated that interaction between Cavin1 and Caveolin1 displayed a significant role in EV production and function in glioma cells.

Adhering to the principle that structure determines function, a few studies have focused on the structure of Cavin1 protein. Cavin1 exhibits a highly conserved trimeric coiled-coil architecture with a conserved N-terminal region containing heptad repeats of hydrophobic amino acids. Reportedly, the N-terminal leucine-zipper (LZ) motif is essential for localization of Cavin1 in caveolae at the plasma membrane 32, 33. Homology modeling confirmed that although a hydrophobic transmembrane domain (102-134 residue) was found in Caveolin1, no such transmembrane structure was found in Cavin1. Therefore, unlike Caveolin1, Cavin1 cannot directly bind to the plasma membrane and it is more likely that Cavin1 anchors to the membrane by binding with other proteins, such as Caveolin1.

Interaction between Cavin1 and Caveolin1 is crucial for the genesis and function of normal caveolae. Absence of Cavin1 increased the lateral mobility of Caveolin1 oligomers, thereby hindering the formation of stable caveolae. Moreover, Caveolin1 oligomers on the plasma membrane are able to transform from a flat profile to the characteristic caveolar appearance only in the presence of Cavin1 28. However, details of the mode of binding between Cavin1 and Caveolin1 are scant and the role such binding plays in tumor biology is poorly understood. Therefore, we designed a variant of Cavin1 (vCavin1) by fusing a positively charged short peptide “TAT” to the N-terminus of Cavin1 to block its interaction with Caveolin1. First, the differences between Cavin1 and vCavin1 in structure, surface electrostatic distribution and binding force with Caveolin1 were analyzed. Considering the limits of traditional experimentation, we devised a new scheme based on computer simulation, which predicted a stable interaction between Cavin1 and Caveolin1 and an unstable binding between vCavin1 and Caveolin1. This prediction was confirmed by the results of co-immunoprecipitation and fluorescence colocalization. Although eGFP-vCavin1 did not bind with Caveolin1, it exhibited substantial membrane localization. However, binding with Caveolin2 alone was not sufficient to cause such extensive membrane localization. More investigations are needed to elucidate the process by which eGFP-vCavin1 anchors to the cell membrane. Nevertheless, we demonstrated that blocking the interaction between Cavin1 and Caveolin1 not only eliminated Cavin1-induced increasing EV secretion, but also excluded Cavin1 from EVs, indicating that the protein was loaded onto EVs not randomly, but selectively.

Exploration of molecular mechanisms regulating production and function of tumor-derived EVs, with particular reference to communication within the tumor microenvironment, is in its initial stages. Previous proteomic studies indicated that EVs contain a specific series of proteins from endosomes, the plasma membrane and the cytosol, while few from other organelles 34. There are two known genesis routes for EVs: (i) inward budding inside endosomes which mainly originate from the endocytic system; and (ii) budding directly from the plasma membrane. Mechanisms underlying the regulation of EV genesis and secretion by proteins, including RAB GTPases (RAB-2B, 5A, 7, 9A, 11A, 27A, 27B, and 35), small GTPase ADP Ribosylation Factor 6 (ARF6) and SNARE proteins (VAMP-7, YKT-6 and Syx-5) have not been well understood 35-41. In fact, it is possible that many factors that regulate EV secretion remain unrecognized. A previous study demonstrated that Cavin1 expression in the prostate cancer cell line, PC3, did not alter EV secretion or distribution, but reduced EV internalization and EV-mediated osteoclastogenesis, probably via altering cargo recruitment 42. However, the current study revealed that Cavin1 not only enhanced EV production in the glioma cell lines, U87 and GL261, but also affected EV cargo and function. The contradiction between these two studies may be due to the fact that Cavin1 plays different roles in the biology of different tumor cell lines. Cavin1 emerges as a protein which, when exogenously expressed in prostate cancer cells, attenuates their aggressiveness 43-45. On the contrary, we have recognized Cavin1 as a biomarker for malignancy and poor prognosis in glioma 18. This study provided more evidence for enhancement of glioma malignancy by Cavin1 overexpression.

The effect exerted by EVs on recipient cells depends on uptake. It is noteworthy that Cavin1 expression not only affected EV production, but also altered EV function, especially enhanced internalization. There are multiple potential routes for EV uptake, such as caveolae-mediated endocytosis, clathrin-dependent endocytosis, micropinocytosis or phagocytosis 46-49. However, many questions such as those related to the specificity of EV uptake as well as the association between EV uptake and EV size or cargo, remain unresolved. Previous studies have indicated that protein interactions between EVs and recipient cells facilitated endocytosis of EVs 46, 50-52. Knockdown of Caveolin1 in recipient cells led to significantly impaired EV uptake 53, suggesting that caveolae-associated protein interaction may be involved in the EV uptake process. Nevertheless, the possibility that Cavin1 overexpression in EVs may increase caveolae-mediated EV entry needs further supporting evidence.

Glioma cells secrete EVs containing proteins (EGFR/EGFRvIII, MGMT and APNG) and RNAs (miR-21, miR-23, miR-29a, miR-30a, miR-221 and miR-451) to regulate the proliferation of recipient cells via several signaling pathways (AMPK, AKT, and MAPK) 54-57. Although the molecular mechanism underlying the association between enhanced proliferation of recipient LN229 cells and Cavin1 expressing EVs, remains unclear, it is surmised that Cavin1 and downstream molecules may regulate key factors related to proliferation, via the dual role played by Cavin1 as a caveolae-related protein as well as a transcription factor 58, 59. Furthermore, increased accumulation of Cavin1-overexpressing U87-EVs in glioma might suggest that the permeability of U87-C-EVs across the blood-brain barrier (BBB) was elevated. Endothelial cells in the central nervous system (CNS) which form tight junctions and exhibit unusually low levels of transcytosis to limit permeability, are a core composition of the BBB 60-63. It has been demonstrated that the caveolae-mediated transcytosis in CNS endothelial cells is reduced, compared with that in peripheral endothelial cells 64. Therefore, it was speculated that increased permeability of U87-C-EVs was induced by increased caveolae-mediated transcytosis. On the other hand, efficient uptake of U87-C-EVs by glioma cells might contribute to increased accumulation of U87-C-EVs in glioma.

Glioma recruits and activates microglia, including brain-resident microglia and infiltrating macrophages, to support and promote tumor proliferation and invasion 65, 66. These microglia/macrophages, also termed as tumor-associated macrophages (TAM), are categorized into 2 subtypes according to different activation patterns: (i) classically activated M1 phenotype; and (ii) alternatively activated M2 phenotype 67-69. Generally, pro-inflammatory M1 is regarded as a tumor-suppressive phenotype whereas M2 is considered as a tumor-supportive type 8, 70. However, studies have revealed that M1 specific markers are positively correlated with glioma growth and progression 71-73. A recent study reported that hypoxic glioma-derived EVs induced M2 macrophage polarization 74. The results of the current study indicated that both M1 and M2 markers were elevated in Cavin1 overexpression group, suggesting that Cavin1 overexpressing glioma cells exerted a general activating effect on microglia/macrophages via EVs. On one hand, the exact composition of TAMs in glioma changes with time 75, 76. On the other hand, the current dichotomous classification of TAMs may be too simplified to accurately define TAMs of all function states, and communication between glioma cells and TAMs may be much more complicated and diversified than expected. Therefore, the dynamic activation state and function of these glioma-TAMs, and the detailed roles played by Cavin1-expressing glioma-EVs in TAM reprogramming need further investigation.

In conclusion, we demonstrated that Cavin1 overexpression in glioma cell lines, U87 and GL261, not only enhanced EV production, uptake and homing ability, but also promoted EV mediated proliferation of nearby glioma cells and the recruitment and activation of microglia/macrophages. However, above results were not observed in vCavin1 expressing glioma cells, suggesting an important role for Cavin1-Caveolin1 interaction in the production as well as function of EVs. Further investigation is needed to clarify the critical question of which molecules and pathways besides Caveolin1 are involved in the Cavin1-mediated effect on glioma-derived EVs. Our study provides a potential theoretical interpretation for the positive correlation between Cavin1 expression and poor prognosis in glioma patients 18. Moreover, our findings indicated a promising therapeutic target for those gliomas that express high levels of Cavin1.

## Supplementary Material

Supplementary figures and tables.

## Figures and Tables

**Table 1:** Comparison of Cavin1 and high homology template sequences

RANK	PDB HIT	Ident1	Ident2	Cov	Norm. Z-score
1	5H7CA	0.12	0.20	0.98	1.17
2	1VW1A	0.11	0.19	0.98	1.41
3	4QKWA	0.44	0.11	0.26	1.54
4	4UXVA	0.09	0.20	1.00	2.06
5	4QKVA	0.44	0.11	0.26	7.76

**Table 2:** Comparison of vCavin1 and high homology template sequences

RANK	PDB HIT	Ident1	Ident2	Cov	Norm. Z-score
1	4UXVA	0.11	0.21	0.99	1.06
2	6H2XA	0.09	0.16	0.80	1.46
3	4QKWA	0.44	0.11	0.25	1.61
4	4QKVA	0.44	0.11	0.25	5.56
5	5YFPE	0.09	0.18	1.00	1.67

**Table 3:** The telechelic cavity depth of Cavin1 and vCavin1, and the distance of the binding site on Caveolin1 to plasma membrane.

Protein	Depth/ Å	Teleclaw gap width / Å	Distance to membrane / Å
Caveolin1	/	/	36.0
Cavin1	25.5	60.0/52.1/35.0	/
vCavin1	52.6	52.2/41.8/33.7	/

**Table 4:** Hydrogen bond interaction analysis on Caveolin1-Cavin1 (A: Caveolin1, B: Cavin1)

RUN	X─H…Y	Donor Atom	Acceptor Atom	Distance	Angle DHA
**1**	A: MET1:H -B: LYS45:O	H	O	2.6	123.1
**2**	A: SER9:H -B: GLU10:OE1	H	OE1	2.0	132.8
**3**	A: TYR14:OH -B: TYR7:HO	OH	HO	2.7	100.8
**4**	B: ARG11: HO2 -A: GLY22: O	HO2	O	2.6	159.0
**5**	B: ARG11: HE -A: GLY22: O	HE	O	1.9	122.8
**6**	B: ARG11: 1HH1 -A: ASN23:OD1	1HH1	OD1	2.2	152.3
**7**	A: LYS26:1HZ -B: TYR7: O	1HZ	O	2.4	131.9
**8**	A: LYS26:3HZ -B:GLU10:OE2	3HZ	OE2	1.9	111.8
**9**	A: ASN28:1HD2 -B: ASP3:OD2	1HD2	OD2	1.7	96.4
**10**	A: ASN29:1HD2 -B: GLU10:OE1	1HD2	OE1	1.9	108.0
**11**	B: TYR7:OH -A: GLU35:OE2	OH	OE2	1.8	102.5
**12**	B: ASN50: H -A: GLN40:OE1	H	OE1	2.5	130.8
**13**	A: LYS47: 2HZ -B: ASP66:OD2	2HZ	OD2	2.5	138.4

**Table 5:** Hydrogen bond interaction analysis on Caveolin1-vCavin1 (A: Caveolin1, B: vCavin1)

RUN	X─H…Y	Donor Atom	Acceptor Atom	Distance	Angle DHA
**1**	B:ARG24:2HH1-A:GLU10:OE2	2HH1	OE2	2.1	115.6
**2**	B: ARG12:1HH2-A:ASN29:O	1HH2	O	2.7	107.8
**3**	B: ARG9:1HH2-A: ASP67:OD1	1HH2	OD1	1.8	95.7
**4**	B: ARG5:1HH2-A:ASP70:OD2	1HH2	OD2	2.3	127.2
