# Supplementary material for: Homotrimer cavin1 interacts with caveolin1 to facilitate tumor growth and activate microglia through extracellular vesicles in glioma
Source: Theranostics. 2020 May 17;10(15):6674–94. doi: 10.7150/thno.45688 (PMC7295042; doi:10.7150/thno.45688)
Supplement: Supplementary file 1 — Supplementary figures and tables. [file thnov10p6674s1.pdf]

Supplementary Figures

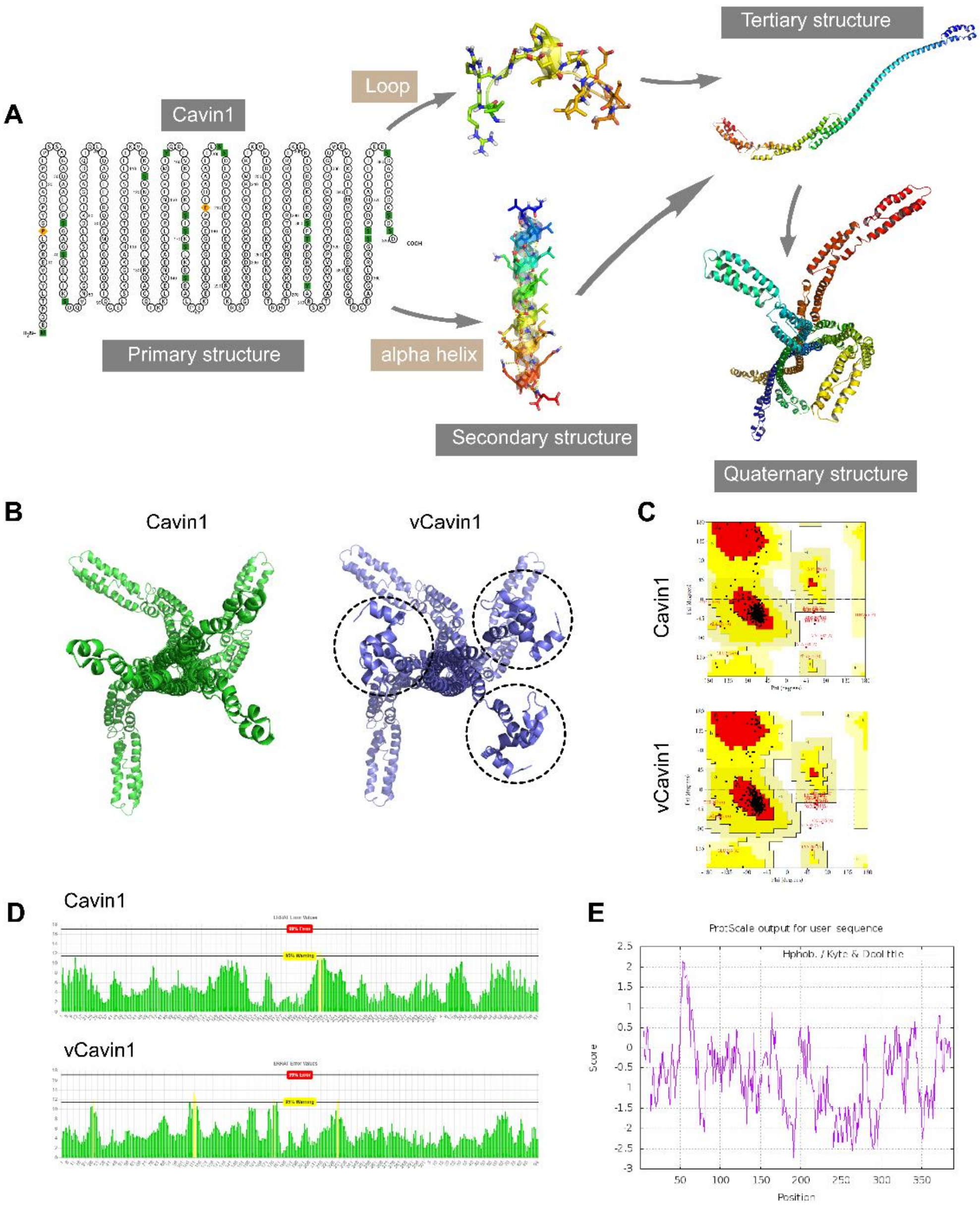

Figure S1. The 3D structure prediction of Cavin1 and vCavin1.

(A) A schematic diagram describing the prediction process. Based on the amino acid sequence of Cavin1, the secondary structure (mainly α-helixes and loops), the tertiary structure (the arrangement of multiple secondary structural elements in three-dimensional space), and the quaternary structure (functional protein complexes formed by interactions between subunits) were predicted in silico. (B) Front view of Cavin1 and vCavin1 trimer. The N-terminus of vCavin1 which is different from that of Cavin1 is circled by the black dotted line. (C) Ramachandran plot of Cavin1 and vCavin1. For Cavin1 and vCavin1 models, the percentage of dihedral angles within a reasonable range was 98.0 % and 98.3 %, respectively. It suggested that the 3D structures of the target protein models were reasonable and accurate. (D) ERRAT plot of Cavin1 and vCavin1. The ERRAT values of Cavin1 and vCavin1 were 99.2147 % and 97.3333 %, respectively, which are relatively close to those of high-resolution crystal structures, indicating the accuracy of the models. (E) Cavin1 protein heavy chain hydrophobicity prediction analysis (> 0 represents hydrophobicity; <0 represents hydrophilicity). The content of hydrophilic residues in Cavin1 was very high (about 93.3 %), showing great hydrophilicity.

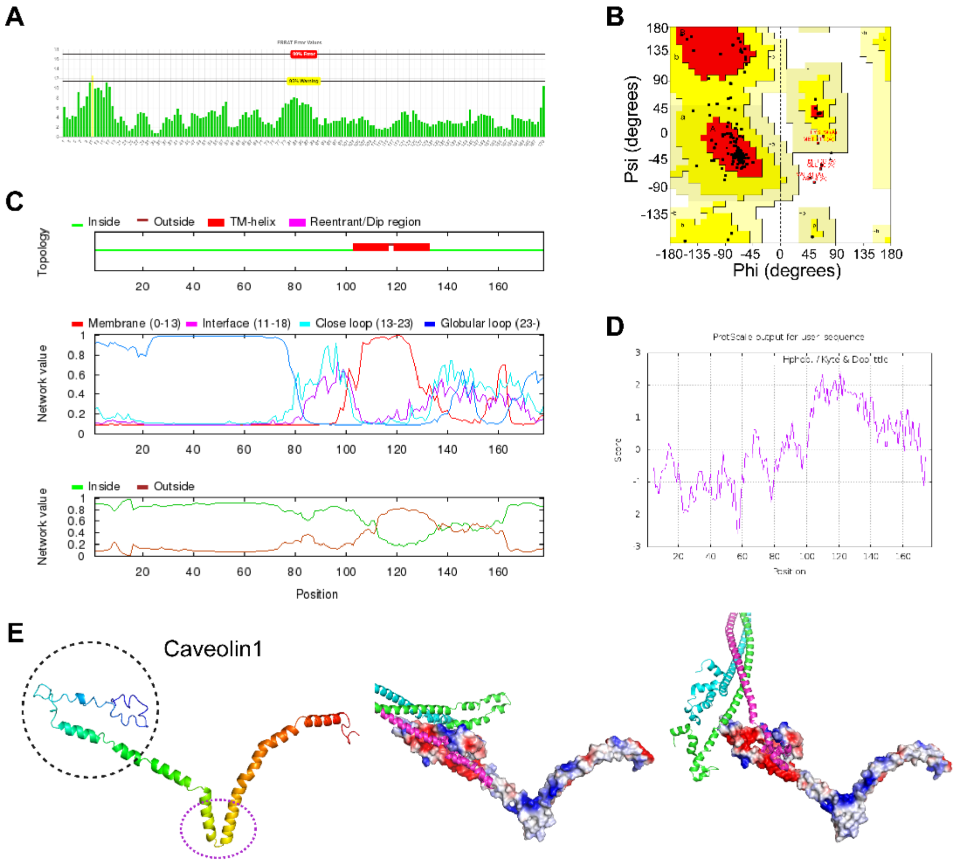

Figure S2. The 3D structure prediction of Caveolin1.

(A) ERRAT plot of Caveolin1. The ERRAT value of Caveolin1 was 99.412 %, indicating a relatively high accuracy of modeling. (B) Ramachandran plot of Caveolin1 showing that the percentage of dihedral angles within a reasonable range was 97.5 %, which indicated a reasonable modeling of Caveolin1. (C) Prediction of the transmembrane structure of Caveolin1 by OCTOPUS. Caveolin1 contains two transmembrane segments (102-117 and 119-133), which form a hairpin structure and are embedded in the plasma membrane. (D) Caveolin1 protein heavy chain hydrophobicity prediction analysis (> 0 represents hydrophobicity; <0 represents hydrophilicity); 47.2% of the residues in Caveolin1 are hydrophilic residues, and N-terminal domains (residues 1-81) were almost all hydrophilic residues, showing great hydrophilicity. It provides the possibility for a hydrogen-bonding interaction network, thereby promoting interaction between Caveolin1 and other proteins and ensuring stable signal transmission. The C-terminal domain including 135-178 residues is a hydrophobic and helical conserved region. (E) 3D structure of full length Caveolin1 and the interaction between Caveolin1 and Cavin1/vCavin1. The transmembrane domain is circled with the purple dotted line and the N-terminus is circled with a black dotted line.

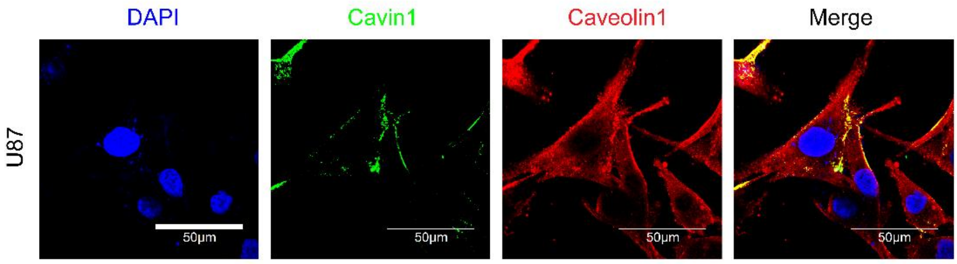

Figure S3. Confocal immunofluorescence imaging of endogenous Cavin1 and Caveolin1 in U87 showing colocalization of endogenous Cavin1 with Caveolin1.

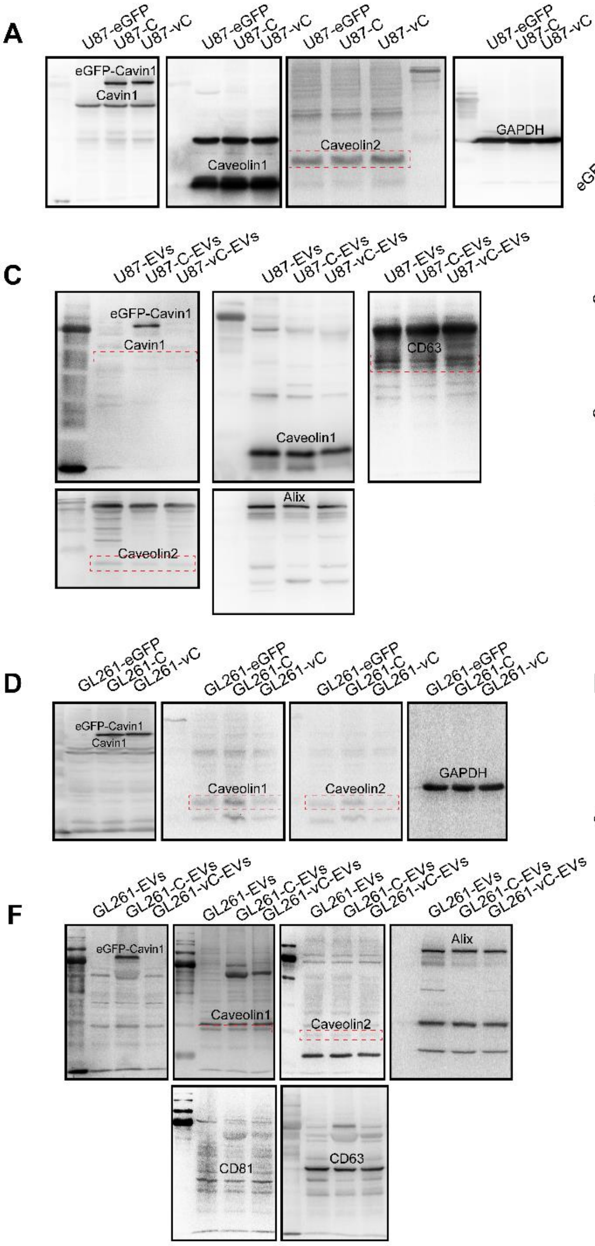

Figure S4. The original pictures of the WB and IP-WB analysis.

(A) WB images showing the expression of eGFP-Cavin1/vCavin1, endogenous Cavin1, Caveolin1 and Caveolin2 in U87-eGFP, U87-C and U87-vC cells. (B) IP-WB analysis detecting the interaction of eGFP-Cavin1/eGFP-vCavin1 with Caveolin1 and Caveolin2 in U87 cells. (C) WB images showing the expression of eGFP-Cavin1/vCavin1, endogenous Cavin1, Caveolin1 and Caveolin2 in U87-EVs, U87-C-EVs, and U87-vC-EVs. (D) WB images showing the expression of eGFP-Cavin1/vCavin1, endogenous Cavin1, Caveolin1, and Caveolin2 in GL261-eGFP, GL261-C, and GL261-vC cells. (E) IP-WB analysis detected interaction of eGFP-Cavin1/eGFP-vCavin1 with Caveolin1 and Caveolin2 in GL261 cells. (F) WB images showing the expression of eGFP-Cavin1/vCavin1, endogenous Cavin1, Caveolin1 and Caveolin2 in U87-EVs, U87-C-EVs and U87-vC-EVs.

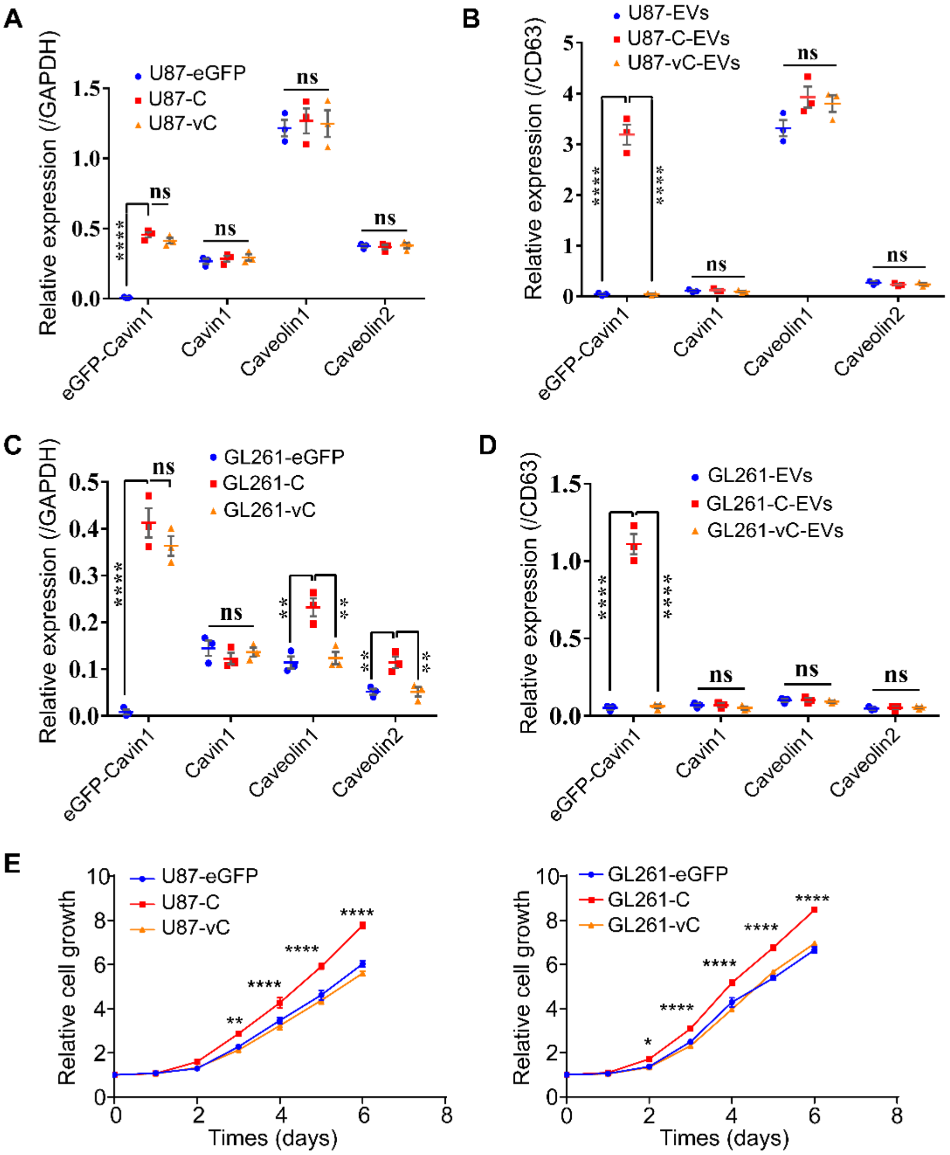
A
Relative expression (/GAPDH)
U87-eGFP
U87-C
U87-vC
eGFP-Cavin1
Cavin1
Caveolin1
Caveolin2
B
Relative expression (/CD63)
U87-EVs
U87-C-EVs
U87-vC-EVs
C
GL261-eGFP
GL261-C
GL261-vC
D
GL261-EVs
GL261-C-EVs
GL261-vC-EVs
E
Relative cell growth
Times (days)
GL261-eGFP
GL261-C
GL261-vC
ns
****
**
*

Figure S5. Quantification of protein expression levels and cell proliferation.

(A) The relative expression of eGFP-Cavin1/vCavin1, endogenous Cavin1, Caveolin1 and Caveolin2 in U87-eGFP, U87-C and U87-vC cells. eGFP-Cavin1/vCavin1: U87-C *vs* U87-eGFP, $p < 0.0001$; U87-C *vs* U87-vC, $p > 0.05$. Cavin1: $p > 0.05$. Caveolin1: $p > 0.05$. Caveolin2: $p > 0.05$. (B) The relative expression of eGFP-Cavin1/vCavin1, endogenous Cavin1, Caveolin1 and Caveolin2 in U87-EVs, U87-C-EVs and U87-vC-EVs. eGFP-Cavin1/vCavin1: U87-C-EVs *vs* U87-EVs, $p < 0.0001$; U87-C-EVs *vs* U87-C-EVs, $p < 0.0001$. Cavin1: $p > 0.05$. Caveolin1: $p > 0.05$. Caveolin2: $p > 0.05$. (C) The relative expression of eGFP-Cavin1/vCavin1, endogenous Cavin1, Caveolin1 and Caveolin2 in GL261-eGFP, GL261-C and GL261-vC cells. eGFP-Cavin1/vCavin1: GL261-C *vs* GL261-eGFP, $p < 0.0001$; GL261-C *vs* GL261-vC, $p > 0.05$. Cavin1: $p > 0.05$. Caveolin1: GL261-C *vs* GL261-eGFP, $p < 0.01$; GL261-C *vs* GL261-vC, $p < 0.01$. Caveolin2: GL261-C *vs* GL261-eGFP, $p < 0.01$; GL261-C *vs* GL261-vC, $p < 0.01$. (D) The relative expression of eGFP-Cavin1/vCavin1, endogenous Cavin1, Caveolin1 and Caveolin2 in GL261-EVs, GL261-C-EVs and GL261-vC-EVs. eGFP-Cavin1/vCavin1: GL261-C-EVs *vs* GL261-EVs, $p < 0.0001$; GL261-C-EVs *vs* GL261-vC-EVs, $p < 0.0001$. Cavin1: $p > 0.05$. Caveolin1: $p > 0.05$. Caveolin2: $p > 0.05$. (E) Relative growth of U87-eGFP, U87-C, U87-vC, and GL261-eGFP, GL261-C and GL261-vC cells detected using CCK8. At day 6, the relative growth of U87-C was higher than U87-eGFP and U87-vC ($p < 0.0001$, $p < 0.0001$). At day 6, the relative growth of GL261-C was higher than GL261-eGFP and GL261-vC ($p < 0.0001$, $p < 0.0001$).

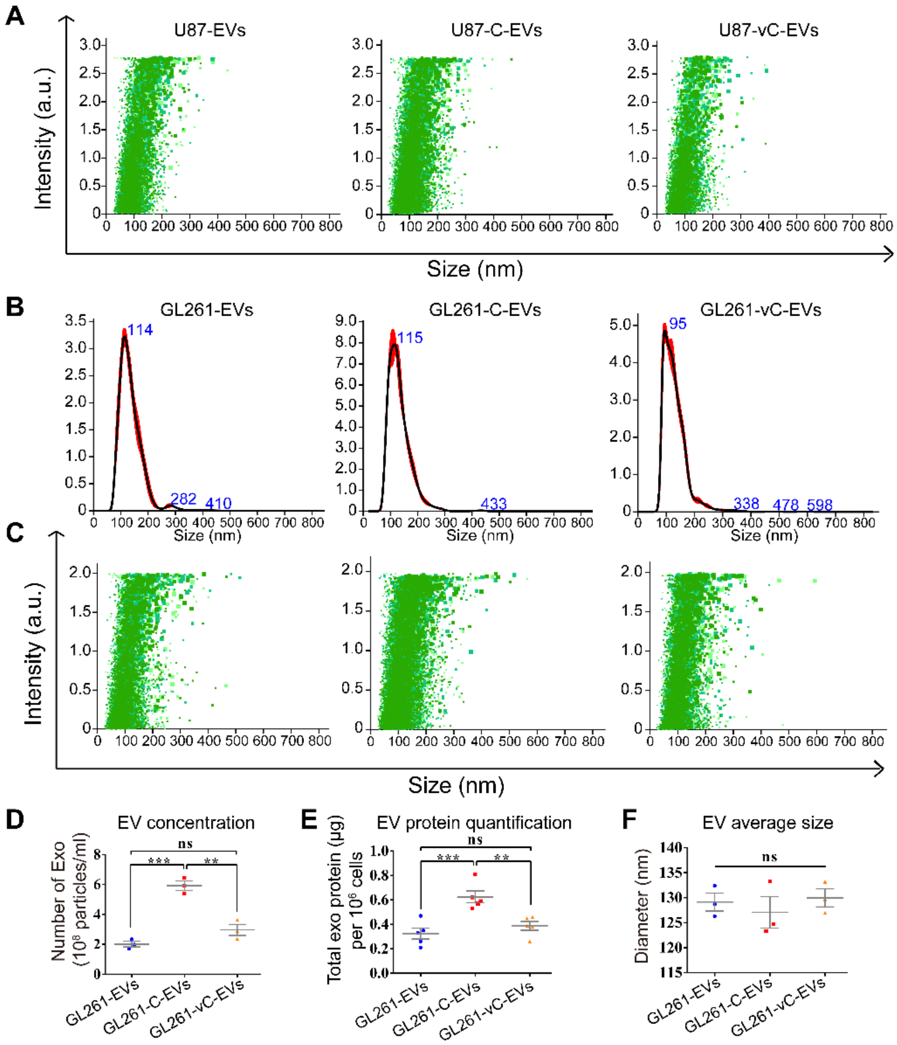

Figure S6. NTA analysis and quantification of EVs.

(A) NTA Intensity/Size graph for U87-EVs, U87-C-EVs and U87-vC-EVs. (B) NTA Concentration/Size graph for GL261-EVs, GL261-C-EVs and GL261-vC-EVs. (C) NTA Intensity/Size graph for GL261-EVs, GL261-C-EVs and GL261-vC-EVs. (D) Concentration quantification of GL261-EVs, GL261-C-EVs and GL261-vC-EVs. GL261-C-EVs *vs* GL261-EVs, $p < 0.001$; GL261-C-EVs *vs* GL261-vC-EVs, $p < 0.01$. (E) Protein quantification of GL261-EVs, GL261-C-EVs and GL261-vC-EVs. GL261-C-EVs *vs* GL261-EVs, $p < 0.001$; GL261-C-EVs *vs* GL261-vC-EVs, $p < 0.01$. (F) Quantification of the average size of GL261-EVs, GL261-C-EVs and GL261-vC-EVs. $p > 0.05$.

Supplementary Tables

Table S1. Number and proportion of amino acids of Cavin1

Amino acids	Number (content)
Glu (E)	56（14.4%）
Lys (K)	39（10.0%）
Leu (L)	38（9.7%）
Val (V)	33（8.5%）
Ala(A)	32（8.2%）
Arg (R)	29（7.4%）
Ser (S)	29（7.4%）
Gly (G)	23（5.9%）
Asp (D)	21（5.4%）
Thr (T)	19（4.9%）
Pro (P)	15（3.8%）
Gln (Q)	13（3.3%）
Ile (I)	13（3.3%）
Asn (N)	8（2.1%）
Met (M)	6（1.5%）
Phe (F)	6（1.5%）
His (H)	5（1.3%）
Tyr (Y)	5（1.3%）

Table S2. Physicochemical properties of the heavy chain of Cavin1

Physicochemical properties	Myosin heavy chain
Atomic number	6167
Number of amino acid residues	390
Molecular formula (molecular weight)	$C_{1879}H_{3125}N_{547}O_{619}S_{6}$(43476.14）
Theoretical isoelectric point	5.5
Number and ratio of negatively charged residues (Asp+Glu)	77（19.7%）
Number and ratio of positively charged residues (Arg+His+Lys)	73（18.7%）
Number of sulfydryl	0
Half-life period（h）	30
Extinction coefficient	7450
Instability index (>40, indicating protein instability; <40, indicating protein stability)	40.75
Fat coefficient	83.74
Total average hydrophilicity (>0, indicating hydrophilic; <0, indicating hydrophobic)	-0.745
